# Foot-and-Mouth Disease Virus: Molecular Interplays with IFN Response and the Importance of the Model

**DOI:** 10.3390/v14102129

**Published:** 2022-09-27

**Authors:** Morgan Sarry, Damien Vitour, Stephan Zientara, Labib Bakkali Kassimi, Sandra Blaise-Boisseau

**Affiliations:** 1 UMR VIROLOGIE, INRAE, École Nationale Vétérinaire d’Alfort, ANSES Laboratoire de Santé Animale, Université Paris-Est, 94700 Maisons-Alfort, France; 2 AgroParisTech, 75005 Paris, France

**Keywords:** protein–protein interactions, virus–host interactions, foot-and-mouth disease virus, interferon response, study models

## Abstract

Foot-and-mouth disease (FMD) is a highly contagious viral disease of cloven-hoofed animals with a significant socioeconomic impact. One of the issues related to this disease is the ability of its etiological agent, foot-and-mouth disease virus (FMDV), to persist in the organism of its hosts via underlying mechanisms that remain to be elucidated. The establishment of a virus–host equilibrium via protein–protein interactions could contribute to explaining these phenomena. FMDV has indeed developed numerous strategies to evade the immune response, especially the type I interferon response. Viral proteins target this innate antiviral response at different levels, ranging from blocking the detection of viral RNAs to inhibiting the expression of ISGs. The large diversity of impacts of these interactions must be considered in the light of the in vitro models that have been used to demonstrate them, some being sometimes far from biological systems. In this review, we have therefore listed the interactions between FMDV and the interferon response as exhaustively as possible, focusing on both their biological effect and the study models used.

## 1. Introduction

**Figure 1:**
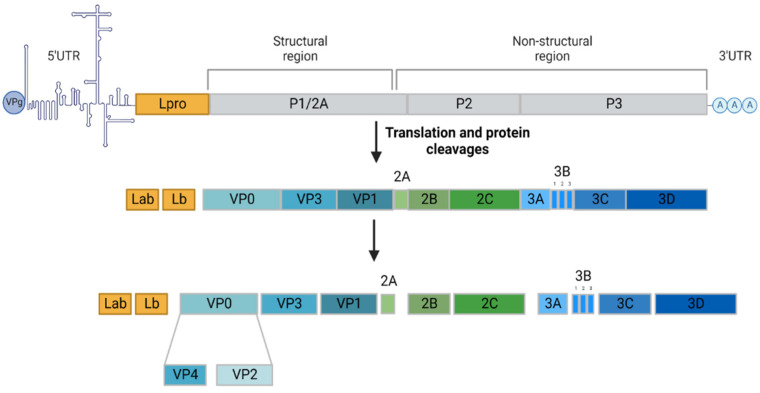
Foot-and-mouth disease virus genome structure and viral protein expression. The FMD virus has a small genome (about 8.5 kb) encoding four structural proteins, VP4, VP2, VP3 and VP1, forming the capsid, and eleven nonstructural proteins, Labpro, Lbpro, 2A, 2B, 2C, 3A, 3B1, 3B2, 3B3, 3C and 3D, responsible for the infectious cycle. Adapted with permission from Belsham et al., 2020 [2]. Created with BioRender.com (accessed on 15 August 2022).

Foot-and-mouth disease (FMD) is a highly contagious disease affecting domestic and wild cloven-hoofed animals, namely productive animals, such as cattle, sheep, goats and swine. It remains a major threat to animal health since, in the event of an epizootic, its impact is considerable from both a societal and economic point of view. The etiological agent responsible for FMD is known as foot-and-mouth disease virus (FMDV). This pathogen is a positive-sense single-stranded RNA virus belonging to the genus *Aphtovirus* within the *Picornaviridae* family. The high mutation rate of the FMDV genome, due to poor fidelity of the RNA replication and lack of proofreading activity has led to the classification of the FMDV into seven different serotypes, namely A, O, C, Asia 1, South African Territories 1 (SAT1), SAT2 and SAT3, each subdivided into several subtypes. The antigenic diversity among these serotypes constitutes an obstacle to vaccine efficiency since there is no cross-protection between them [1]. The viral RNA genome is approximately 8 kb in length containing an open reading frame (ORF), flanked by untranslated regions (5′UTR and 3′UTR). The ORF encodes a single polyprotein precursor comprising four structural proteins (VP1, VP2, VP3 and VP4) and eleven nonstructural proteins (Labpro, Lbpro, 2A, 2B, 2C, 3A, 3B1, 3B2, 3B3, 3C and 3D) (Figure 1).

## 2. FMDV Life Cycle

**Figure 2:**
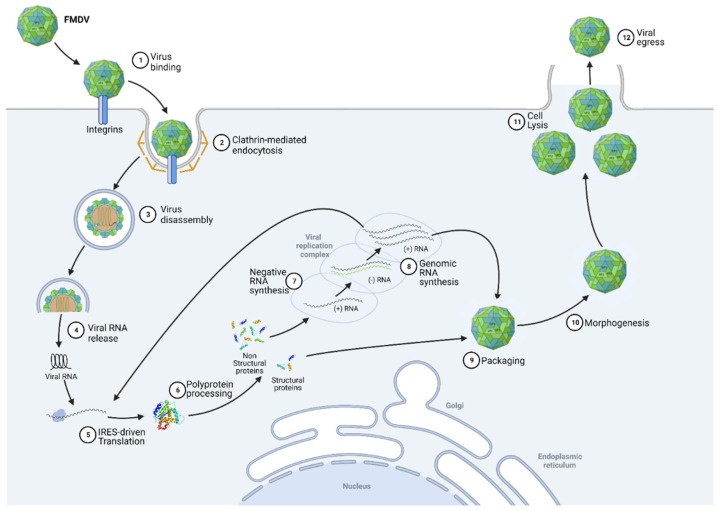
Foot-and-mouth disease virus replication cycle (See text for details). Created with BioRender.com (accessed on 15 August 2022).

The FMDV replication cycle can be characterised by nine distinct phases, namely binding, internalization, uncoating, translation, processing, replication, encapsidation, morphogenesis and viral egress (Figure 2). Infection begins with capsid adsorption to membrane receptors via a highly conserved RGD motif (Arg-Gly-Asp) at the VP1 surface [3]. In most cases, the virus binds to receptors belonging to the integrin family, particularly to αVβ6 integrins that are highly expressed in epithelial cells. Sometimes, FMDV also binds to other receptors, such as heparan sulphate (HS) [4]. Once adsorbed, virions are internalised, usually by clathrin-mediated endocytosis [5]. While the mechanisms of FMDV uncoating are still incompletely understood, it appears that endosomal acidification results in the uncoating of the viral capsid, leading to the release of the genomic RNA into the cytoplasm of the cell [6]. It has been indeed shown that at pH values corresponding to early endosomes, the aphthovirus capsid dissociates into pentameric subunits, releasing RNA and the internal protein VP4 [2,7]. The genomic RNA is then translated, beginning at the internal ribosome entry site (IRES) element, to produce a large polypeptide called precursor polyprotein. Viral genomic RNA is processed into mature polypeptides as well as a variety of partial cleavage intermediates to generate the fifteen final mature viral proteins mediated by the action of the two viral proteinases (Lpro and 3C) [2]. While the FMDV shuts down host translation thanks to Lpro activity, its replication is carried out by the 3D RNA-dependent RNA polymerase, along with the 2B, 2C and 3A proteins, in membrane structures derived from the endoplasmic reticulum (ER) and Golgi [8,9]. A negative intermediate RNA is first synthesised from the positive strand. This antigenomic RNA serves as a template for the synthesis of genomic RNA. The completion of the infectious cycle then requires the assembly of structural proteins, which are organised into pentamers, associating together to form the capsid. The genomic RNA then binds with this immature capsid to form a provirion [10]. A final protein cleavage of the VP0 precursor into VP2 and VP4 then occurs, transforming the provirion into a mature virus particle. The virions rapidly accumulate in the cytoplasm of infected cells, inducing cell lysis and resulting in viral egress of the neoformed viruses.

## 3. FMDV Persistence in Its Hosts

The severity of clinical symptoms associated with FMD differs according to the species affected. However, they usually include fever, sudden lameness, lesions on the hooves, tongue and teats and reduced productivity [11]. After clinical recovery, FMDV persists in more than 50% of ruminants, without clinical signs, irrespective of their specific immune status towards this virus [12]. Persistent FMDV infection is currently defined as the detection of the infectious virus in the oropharynx for more than 28 days after infection [13]. However, this definition is challenged in the light of more recent studies that indicate that in the case of nonpersistent infection, viral clearance occurs within the first 21 days postinfection [14]. The persistence of the FMDV has been demonstrated in cattle and small ruminants but not in swine [15]. Domestic pigs are unlikely to be long-term carriers of the infectious FMDV, although persistent FMDV proteins and RNA are frequently detected after infection in lymphoid tissues [16]. In ruminants, viral proteins and RNA are also detected, in addition to infectious viruses in persistence sites [11,12]. Localisations of viral persistence have been described in several species. The FMDV has been thus detected in bovine nasopharyngeal tissues, particularly at the soft palate dorsal surface and in the adjoining dorsal nasopharynx [17]. In ovine, persistent virus is mainly located in the epithelial crypts within ovine oropharyngeal and laryngopharyngeal tonsils [18]. The anatomic sites of FMDV persistence in African buffalo are the pharyngeal and palatine tonsils as well as the dorsal soft palate [19]. Regarding the fact that they are not likely to excrete infectious virus in their environment, healthy carriers represent a transmission risk that is considered extremely low. However, it has recently been shown that in the case of superinfection, a frequent phenomenon in natural conditions, animals persistently infected can shed the infectious virus [20]. Furthermore, viral recombination phenomena have been evidenced during these experimental superinfections, thereby strengthening the importance of healthy carriers as actors in the emergence and dissemination of new recombinant strains of FMDV. Similarly, quite a high recombination rate could be observed in coinfections in African buffalo, indicating that recombination could play an important and unappreciated role in the evolution of FMDV [21]. These healthy carriers thus represent a significant risk of new variants being transmitted to susceptible animals and therefore constitute an obstacle to the disease’s control.

Although viral persistence was described in the 1950s, the mechanisms for establishing, maintaining and resolving the persistence of the FMDV remain unclear. The establishment of a persistently infected baby hamster kidney (BHK21) cell line has demonstrated coevolution between the FMDV and host cells [22]. As for many other virus–cell systems, this in vitro coevolution is characterised by a set of genetic variations of both virus and cells, leading to an increased resistance of the latter to infection and an increased infectivity of the persistent virus on naive cells. This investigation also revealed mutations in the persistent virus, particularly in the VP1 and VP3 sequences, which could be involved in the induction and/or maintenance of this persistence [23]. This coevolution phenomenon was then confirmed in a study based on a far more appropriate model for natural infection, namely primary cells derived from bovine pharynx, suggesting that viral persistence results from the combination of a highly specialised epithelial cell population and a specific cellular response, probably involving interferons and cytokines. Indeed, in this study, it has been shown that certain cytokines with a key role in the antiviral response are absent in persistent infection [24]. This coevolution between the FMDV and these primary bovine cells was also highlighted by the colocation between VP1 and the autophagy proteins ATG5 and ATG12 implicated in the antiviral response, making possible their potential interaction [24]. Similarly, the FMDV–host coevolution was demonstrated in persistently infected Madin–Darby Bovine Kidney (MDBK) epithelial cell lines. The sequencing of the VP1 protein of the persistent virus revealed a single-point mutation, consisting of an amino acid substitution from valine (V) to alanine (A) at position 50 that could disrupt a predicted SUMO-binding site. As SUMOylation phenomena are known to be exploited by some viruses to evade the immune response, it is possible that the persistent FMDV could also manipulate this pathway [25].

## 4. FMDV and Innate Immunity

**Figure 3:**
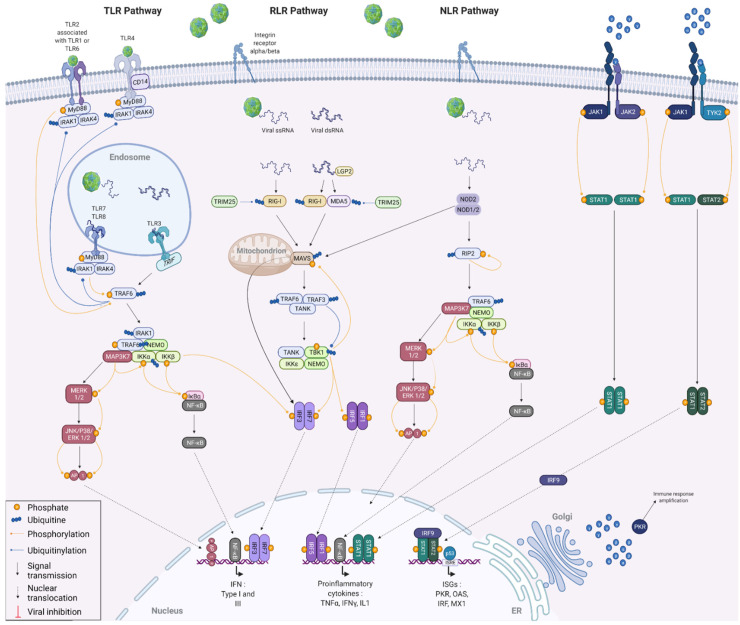
Innate antiviral immune responses against viral infection. The induction of the IFN response results from the recognition of characteristic viral patterns by cellular membranous or cytoplasmic receptors. Activation of these receptors triggers a signalling cascade in the cell, leading to the activation of transcription factors involved in IFN alpha and beta and proinflammatory cytokine production. Finally, the signalling phase consists of the binding of IFN-alpha and -beta to their receptors, which generates an activation signal that is propagated into the cell via the JAK/STAT pathway to enable the expression of numerous proteins with antiviral or immunomodulatory activity. Created with BioRender.com (accessed on 15 August 2022).

In a more general perspective, it is thus conceivable that the FMDV, via a coevolution with its host, has become able to hijack the cellular machinery in order to protect itself against the antiviral response. The coevolution between viruses and their hosts is mainly driven by their opposite but also common interests focused on their respective survival [26]. Indeed, host cells need to detect pathogens efficiently and be able to react against infection. In order to do this, they generally trigger an inflammatory response that is strong enough to eliminate the viruses, but at the same time sufficiently moderate not to affect their own functions. On the virus side, they need to protect themselves from the immune response, without completely overpowering the host to keep it alive [27]. The antiviral innate immunity is largely based on the synthesis and secretion of type I interferons (IFNs) by the infected cells, which induce an antiviral state within infected and surrounding cells [28]. The type I IFN response is characterised by three successive phases, namely induction, production and signalling (Figure 3) [29]. Upon FMDV infection, viral RNA can be detected by cytosolic receptors, such as the retinoic acid-inducible gene I (RIG-I)-like receptors (RLRs) melanoma differentiation-associated gene 5 (MDA5), RIG-I and laboratory of genetics and physiology protein 2 (LGP2), as well as NOD-like-receptors (NLRs) [30]. RIG-I recognises cytoplasmic short double-stranded and 5′-triphosphate single-stranded viral RNAs, while MDA5 senses predominantly long double-stranded RNA [31]. LGP2 acts as a positive regulator of RIG-I- and MDA5-mediated detection of exogenous RNA by complexing with double-stranded viral RNAs generated during the FMDV replication. The interaction between these cytosolic receptors and viral RNA results in the exposure of their CARD domains, which bind to mitochondrial antiviral signalling protein (MAVS) CARD domains by polymerisation. The NLR nucleotide-binding oligomerization domain-containing protein 2 (NOD2), is also able to recognise certain viral recognition patterns, but interacts with MAVS independently of the CARD domains [32]. NOD2 binding to a viral ssRNA also induces its homo- or heterodimerisation with NOD1, allowing receptor-interacting serine/threonine-protein kinase 2 (RIP2) recruitment via an interaction between their CARD domains and promoting its autophosphorylation [33]. Endosomal RNAs are detected by membrane toll-like receptors (TLRs) receptors, such as TLR3, TLR7 and TLR8, while the structural proteins VP1 and VP3 can be recognised by TRL2 and TRL4 receptors, respectively [34,35,36,37]. Upon activation by ligand recognition, these TLRs interact with adaptor proteins, namely TIR domain-containing adapter molecule 1 (TRIF) and myeloid differentiation primary response protein MyD88 (MYD88), itself associated with interleukin-1 receptor-associated kinase 1 (IRAK1) and IRAK4. Interactions between cytoplasmic sensors and MAVS, autophosphorylation of RIP2, as well as conformational changes of TLR-associated adaptor proteins, are responsible for the enrolment of E3 ubiquitin ligases, namely tumour necrosis factor receptor-associated factors 3 and 6 (TRAF3 and TRAF6). These are involved in the polyubiquitination of TANK-binding kinase 1 (TBK1) and the inhibition of nuclear factor kappa-B kinase subunit alpha (IKKα). The polyubiquitin chains are recognised by the NF-kappa-B essential modulator (NEMO), allowing the binding of the attached proteins. The NEMO/TBK1 complex thus forms associates with IKKε and the TRAF family member-associated NF-kappa-B activator (TANK) and induces the phosphorylation of interferon regulatory factors, in particular interferon regulatory factors 3 and 7 (IRF3 and IRF7) [38]. Once phosphorylated, IRFs organize into homo- or heterodimers and translocate to the nucleus [39]. The IKK complex, formed following recognition of polyubiquitinated IKKα by NEMO, is responsible for IκBα phosphorylation. This phosphorylation event causes IκBα degradation and thus nuclear factor NF-kB release which translocates to the nucleus [40]. Similar to the NEMO/TBK1 complex, the IKK complex can phosphorylate IRF7, leading to its dimerization and translocation of the neoformed dimer to the nucleus. In parallel, TRAF6 and NEMO are involved in the recruitment of mitogen-activated protein kinase kinase kinase 7 (MAP3K7), also known as TAK1 [41]. This kinase, which triggers the IKKα phosphorylation needed for NF-kB activation, is also the starting point of the MAPK pathway. This phosphorylation cascade involving various kinases, such as extracellular signal-regulated kinase 1 (ERK1), ERK2, stress-activated protein kinase 1 (JNK1) and JNK2, leads to the phosphorylation of both subunits of activator protein 1 (AP-1). Once phosphorylated, this transcription factor activator translocates to the nucleus.

Once in the nucleus, IRF3 and IRF7 homo- and heterodimers, as well as AP-1, associate with type I IFN promoter to stimulate their transcription. IRF1 and IRF5 dimers are able to boost the transcription of many proinflammatory cytokines, such as TNFα, IFNγ and interleukin IL-1, by binding to their promoters. Similarly, nuclear NF-kB can bind either to the promoter of type I IFNs or to the promoters of proinflammatory cytokines to promote transcription.

When synthesised by the ER, IFNs pass through the Golgi before diffusing into the cellular environment. IFNs can act both autocrine and paracrine. To do so, they are recognised by interferon alpha/beta receptors (IFNAR) [42]. The binding of IFN molecules to these membrane receptors induces the activation of the Janus kinase 1 (JAK1), JAK2 and nonreceptor tyrosine-protein kinase TYK2 proteins [43]. Once activated, these JAK proteins lead to a phosphorylation cascade resulting in the phosphorylation and association of the signal transducer and activator of transcription 1 (STAT1) and STAT2 into homo- or heterodimers [44]. On the one hand, STAT1 homodimers thus assembled translocate to the nucleus where they bind to proinflammatory cytokine promoters to amplify the host inflammatory response. On the other hand, STAT1/STAT2 heterodimers form a complex with IRF9. This complex also translocates to the nucleus and binds specifically to the interferon-stimulated response element (ISRE), present in most IFN-stimulated (ISGs), to induce their expression and activate the antiviral response [27]. The detailed mechanisms involved in the IFN response are illustrated in Figure 2 of this review. Some acute infections can be cleared by the immune system within days, while other infections can be persistent and last for decades. This is because the efficiency of viral clearance depends on many factors, including the immune status of the host, the nature of the virus causing the infection and, most importantly, the virus–host interaction that is established. The IFN response and the resulting ISG expression are thus regulated, even highjacked by the viruses throughout the infection in order to either stimulate it to promote passage into the surrounding cells or to decrease it to avoid being degraded [45].

Among the possibilities explored to explain FMDV persistence, the hypothesis of the modulation of the cellular response by the virus, leading to the establishment and maintenance of a balance between the virus and its host via the establishment of protein–protein interactions (PPI), seems thus interesting. Although many interactions have already been demonstrated between the FMDV and its host in different study models, it is not yet possible to clearly define a relationship between protein interactions and viral persistence [46,47,48]. This can be mainly because most studies of PPI are not performed in a context of acute or persistent infection, but in uninfected cells under overexpression conditions.

It appears interesting to wonder whether the persistence of the FMDV in the host could be explained by the interaction between host and virus proteins. It seems relevant to ask how the virus and its different hosts interact to reach a balanced situation and how these interactions differ between species. It is even possible to wonder whether these differences in PPI could explain why ruminants can become healthy carriers while swine cannot [14,15,16].

**Figure 4:**
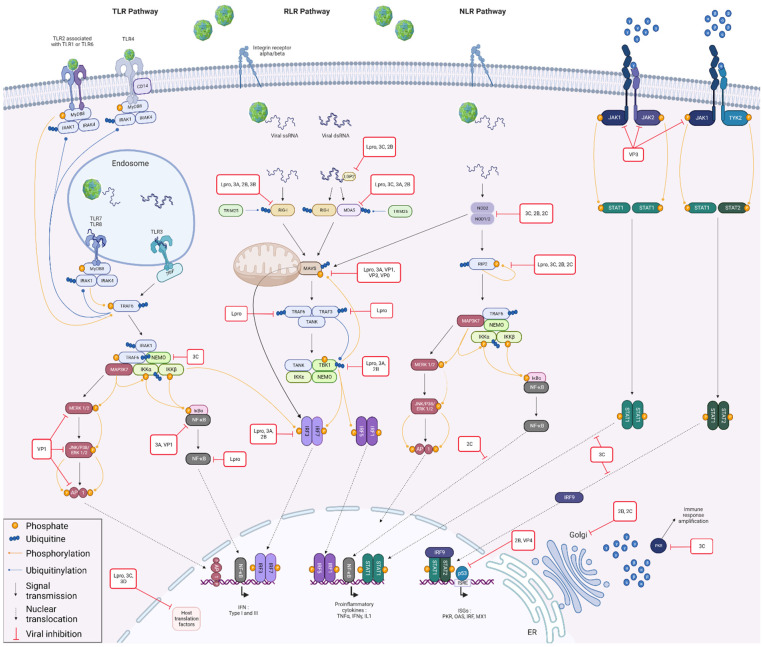
Innate antiviral immune responses and viral counteraction during FMDV infection. Coevolution between FMDV and its hosts has led the virus to adopt a number of strategies for limiting the antiviral immune response. For example, it is able to interfere with viral RNA detection by acting on the RIG-I, MDA5, LGP2 and NOD2 sensors via its Lpro, 2B, 2C, 3A, 3B and 3C proteins. FMDV also antagonises the host immune response by targeting proteins involved in signal transduction, such as MAVS, RIP2, NEMO and TBK1, interacting via its VP0, VP3, VP1, Lpro, 2B, 2C, 3A and 3C proteins. In addition, Lpro protein of FMDV degrades IRF3 and IRF7, while 2B and 3A target only IRF3. MAPK pathway is restricted by the action of VP1, while NF-kB pathway is impacted by VP1, Lpro, 2C and 3A. FMDV also counteracts JAK/STAT pathway via VP3/JAK interaction and by preventing nuclear translocation of STAT dimers through 3C. At a nuclear level, Lpro, 3C and 3D are involved in the inhibition of host translation factors, while VP4 and 2B negatively regulate ISG transcription. Golgi apparatus-associated molecular transport complexes are subverted by 2B and 2C, and some antiviral proteins, such as PKR, are degraded by 3C. Created with BioRender.com (accessed on 15 August 2022).

To answer these questions, it seemed to us relevant to look at the FMDV/host PPI already described in the literature, considering the species used to demonstrate them. This review will provide an overview of the role of each of the FMDV proteins in its escape from the host immune response. The interactions between FMDV and cellular proteins involved in the type I interferon response will be particularly detailed. The viral proteins will be presented in order of relative importance with respect to their involvement in interactions with the IFN pathway. This decision was made in order to avoid the commonly made cleavage between structural and nonstructural proteins. Indeed, recent years have revealed an increasingly more important role for structural proteins in the subversion of the immune response, whereas their contribution was previously rather neglected. All protein interactions, involved in counteracting the immune response, mentioned in this review will be summarised in Table 1. The main ones will be illustrated in Figure 4.

### 4.1. Lpro

Leader protein, also called Lpro, the first protein of the FMDV to be translated, is a papain-like proteinase that releases itself from the polyprotein by cleavage during viral maturation [96]. There are two different forms of the Lpro protein, namely Labpro and Lbpro, depending on the start codon from which translation is initiated. Lbpro, which is shorter by 28 amino acids, is the predominant form in vivo.

Lpro is an important factor of virulence and is involved in many mechanisms against the host innate immune response, acting mainly at the transcription and translation steps [97]. Among the large number of genes regulated by Lpro, many are ISGs and include genes with antiviral activity, cytokines and transcription factors, involved in the regulation of apoptosis or IFN signalling [98]. Numerous interactions have been described between this proteinase and multiple host proteins involved in the IFN signalling pathway, notably in the induction and in the production phases.

The FMDV leader protein plays a major role in inhibiting host mRNA translation, including those of the IFN pathway. Indeed, the Lpro protein counteracts the immune response from the viral RNA sensing phase until the inhibition of the expression of ISGs. Many interactions have been described between this proteinase and multiple host translational factors, leading to the impairment of host mRNA translation and then to the global immune response shut-off [99]. Thus, it has been shown that Lpro directly cleaves human eukaryotic translation initiation factors 4 gamma I and II (eIF4G-I and eIF4G-II) [58,100]. Similar to human rhinovirus (HRV) infection, the Lpro cleavage of both eIF4G-I and eIF4G-II occurs very early in infection, simultaneously and can be achieved in the absence of viral RNA replication within infected cells [101]. eIF4G-I and eIF4G-II are components of the complex eukaryotic translation initiation factor 4F (eIF4F), which is involved in the recognition of the mRNA cap, ATP-dependent unwinding of 5′-terminal secondary structure and recruitment of mRNA to the ribosome [102]. As the FMDV initiates translation in a cap-independent way through the action of the IRES, the virus translation is not dependent on eIF4G-I and eIF4G-II [103]. Likewise, the Lpro cleavage of human polypyrimidine tract-binding protein (PTB), an RNA-binding protein that binds to intronic polypyrimidine tracts to negatively regulate exons splicing, also contributes to the translation shut-off in infected cells [57].

Lpro also cleaves the human gem-associated protein 5 (Gemin 5) at the RGRAR motif, a sequence present in several RNA-binding proteins. Gemin 5 is an essential protein for the formation of the survival of motor neuron (SMN) complex, which is involved in the assembly of small nuclear ribonucleoproteins (snRNPs) forming the spliceosome. The interaction between Lpro and Gemin 5 thus disrupts the proper splicing of cellular premRNAs [59]. The Lpro processing of human eukaryotic translation initiation factor 3 subunits a and b (eIF3a and eIF3b), RNA-binding components of the eukaryotic translation initiation factor 3 (eIF3) complex, prevents the assembly of 48S initiation complexes on host mRNAs [104].

The proteolytic cleavage of human polyadenylate-binding protein (PABP) inhibits its binding to the poly-A tail of mRNA and then the mediated-PABP regulation processes of mRNA metabolism, such as premRNA splicing and mRNA stability RNA binding [57]. Thus, the cleavage of these host factors allows the FMDV to make full use of the host cell machinery for viral protein synthesis [105].

In addition, Lpro is responsible for the degradation of accumulated p65 in goats, a subunit of the transcription factor nuclear factor kappa B (NF-κB), which contains a DNA-binding domain and a transactivation domain, responsible for the transcriptional regulatory activities of NF-κB. This interaction between Lpro and p65 prevents the activation and translocation of NF-κB through the canonical pathway, and thus inhibits the expression of many NF-κB-dependent proinflammatory cytokines [55].

This viral protein can also efficiently reduce the production of interferons by blocking the induction of genes whose transcription depends on IRFs. Indeed, Lpro is responsible for the proteolytic degradation of porcine IRF3 and IRF7 in a dose-dependent manner, but does not affect their mRNA transcription [54]. IRF3 is a regulator of type I IFN gene transcription, particularly activating IFN-β production during the early and late phases of induction. Its phosphorylation by the inhibitor of nuclear factor kappa-B kinase subunit epsilon (IKKε) or TBK1 is responsible for a conformational change. This is followed by homo- or heterodimerization with IRF7, leading to the translocation of the dimers into the nucleus and allowing the transcription of the IFN genes [106]. IRF7 is also a regulator of type I IFN gene transcription that is mainly involved in the late phases of induction. Its activation is similar to IRF3, but unlike IRF3, this protein can activate IFN-β as efficiently as IFN α production after activation by the TLR or RLR pathways [38]. Additionally, it may have a role in regulating the adaptive immune response by inducing proteasome activation [107]. IRF3 and IRF7 are also involved in the activation of the ISG transcription by binding to an ISRE in their promoters. Similar observations have been reported in many picornaviruses. As an example, Theiler virus Lpro is able to block the translocation of mouse IRF3 to the nucleus, preventing its binding to IFN-α/β promoters [108]. Likewise, mengovirus Lpro blocks the induction of IFN-α/β by preventing both NF-κB and IRF3 activity in mouse cells, while Seneca Valley virus 3C pro degrades human IRF3 and IRF7 to abrogate IFN production [109,110].

Lpro binds to human activity-dependent neuroprotector homeobox protein (ADNP), a neuroprotective protein that also has immunomodulatory activity [56]. The purpose of this interaction is to facilitate the ADNP-mediated downregulation of the pro-inflammatory cytokines TNF-α, IL-6 and IL-12 expression to optimise FMDV replication [111].

In order to prevent the detection of viral RNA, Lpro acts early on the cytoplasmic sensors. Thus, Lpro blocks the activation of the immune response mediated by RIG-I, by acting as a deubiquitination enzyme (DUB) that cleaves ubiquitin chains [52]. RIG-I is a receptor that senses cytoplasmic short double-stranded and 5′-triphosphate single-stranded viral RNAs and initiates the signalling cascade leading to the production of type I interferons and proinflammatory cytokines [30]. Upon RNA binding, the Lys 63 polyubiquitination of RIG-I, by the E3 ubiquitin ligase TRIM25, is necessary to bind to the MAVS CARD domain. The transfer of RIG-I to the mitochondrial membrane, via a translocation complex formed by the chaperone protein 14-3-3ε, mediates the association of the RIG-I and MAVS CARD domains, inducing the activation of MAVS, a protein that triggers the downstream signalling cascade [112]. Lpro also inhibits the activation of MAVS mediated by the human MDA5 [51]. Indeed, the viral proteinase cleaves MDA5 at the RGRAR motif, preventing its binding to the CARD domain of MAVS. MDA5 is a member of the retinoic acid-inducible type I receptor family. Like RIG-I, this protein is a cytoplasmic sensor of viral RNA playing a major role in sensing viral infection. Unlike RIG-I, MDA5 recognises long double-stranded RNA. The activation of MDA5 also requires its polyubiquitination, by tripartite motif containing 25 (TRIM25), at Lys 743 [113]. However, it is interesting to note that, contrary to the case of RIG-I, no evidence was found for a possible deubiquitination of MDA5 by the FMDV [50].

In addition, it was established that Lpro is able to cleave human and swine LGP2, an RNA helicase that is also part of RLRs [49]. LGP2 cannot initiate antiviral signalling as it lacks the CARD domain required for activating MAVS-dependent signalling events, but it acts as a regulator of RIG-I- and MDA5-mediated antiviral signalling [114]. For this purpose, LGP2 forms a ribonucleoprotein complex with double-stranded viral RNA generated during picornavirus replication. The formation of this complex induces the recruitment of the E3 ubiquitin-protein ligase RNF135, which improves the detection of exogenous RNA by RIG-I and MDA5 [115]. The cleavage of LGP2 at the RGRAR sequence by Lpro, as with MDA5, results in the formation of truncated proteins that will accumulate in cytoplasm. As these cleavage products are inactive, the efficiency of the antiviral signalling is drastically reduced. While the cleavage of LGP2 by Lpro has also been reported during infection with the Equine Rhinitis A Virus (ERAV), it is interesting to notice that no evidence of this Lpro-LGP2 interaction has been found in other picornaviruses similar to the FMDV, such as the encephalomyocarditis virus (EMCV) or swine vesicular disease (SVDV). Since Lpro interacts with RIG-I, MDA5 and LGP2, the FMDV is able to prevent the antiviral response induced by several proteins individually as well as synergistically, giving the virus a clear advantage for replication and spread in the host.

Although no direct interaction between Lpro and swine RIG-I or MDA5 proteins has been demonstrated, this viral protein inhibits the IFN-λ1 expression pathway induced by RIG-I and MDA5 in pigs. Moreover, the catalytic activity and SAP domain of Lpro have been described to be necessary for suppressing dsRNA-induced IFN-λ1 expression [116]. IFNλ1 or type III interferons, also known as interleukin 28 (IL-28), share many biological characteristics with type I IFNs. Indeed, they are also produced as a result of the activation of the RLR pathway. Like type I IFNs, they enable an antiviral response in the cell and surrounding cells. However, there are differences since dendritic cells and macrophages mainly produce them, and IFNλs bind to their own receptor, which is therefore different from that of IFNαβ [28].

Lpro not only acts on the sensors responsible for triggering the IFN response. Indeed, this viral protein also counteracts proteins further down the signalling cascade to suppress IFN-α/β both at the mRNA and at the protein levels [117]. This is particularly the case for porcine MAVS, which is degraded by Lpro indirectly and by a mechanism that remains unknown to date. Although MAVS levels progressively decreased over time, no cleavage products nor signs of deubiquitination could be observed in FMDV-infected cells [50]. Peroxisomal and mitochondrial MAVS are necessary components of the innate immune defence against viruses. During viral infection, peroxisomal MAVS induces a rapid expression of defence factors that provide short-term protection, while mitochondrial MAVS activates an interferon-dependent signalling pathway that amplifies the immune response. Thus, the Lpro degradation of MAVS prevents its binding to TRAF3 and TRAF6 and to TRAF family member-associated NF-kappa-B activator (TANK) through its pattern recognition receptor (PRR) domain, and then it blocks the further development of the RLR pathway [106].

Lpro also cleaves swine RIP2. After recruitment by NOD2 and autophosphorylation, RIP2 undergoes polyubiquitination, which is necessary for the recruitment of NEMO and MAP3K7, whose activation triggers the signalling cascade responsible for NF-kB activation [53].

MAVS-activated human proteins, such as TRAF3 and TRAF6, are other targets of Lpro [52]. These E3 ubiquitin ligases mediate the synthesis of ‘Lys-63’-linked polyubiquitin chains, which are recognised by proteins, such as NEMO and IRAK1. The binding of the polyubiquitin sensor domains of NEMO and IRAK1 to the ubiquitin chains allows the formation of protein complexes essential for IFN synthesis. The deubiquitination of TRAF3 and TRAF6 by Lpro thus leads to the inhibition of the formation of these protein complexes and to the proper functioning of the RLR pathway.

Lpro also deubiquitinates human serine/threonine-protein TBK1. TBK1 has a key role in the RLR pathway since, following its polyubiquitination by TRAF3 or TANK, its ubiquitin proteins are recognised by the polyubiquitin sensor domain of NEMO. As a result, TBK1 phosphorylates MAVS, leading to the recruitment of IRF3, which is then also phosphorylated by TBK1. Then, the deubiquitination of TBK1, TRAF3 and TRAF6 is an important mechanism in the Lpro-mediated suppression of the signal transduction of the RLR pathway. Since ubiquitination mechanisms are very important in antiviral signal transduction, many viruses have coevolved by developing deubiquitinase activities [118]. The case of FMDV is therefore far from being an exception. As an example, the porcine reproductive and respiratory syndrome (PRRS) protein Nsp2 inhibits NF-κB activation by acting on the ubiquitin chain of nuclear factor of kappa light polypeptide gene enhancer in B-cells inhibitor, alpha (IκBα), thus preventing its degradation [119]. The papain-like protease (PLpro) of severe acute respiratory syndrome coronavirus (SARS-CoV) also has deubiquitinase activity that is involved in subverting the immune response [120]. There are also a few similar cases of the deubiquitination phenomenon within the picornaviruses in which interactions have also been reported for the 3C protease of Seneca Valley virus (SVV), causing the deubiquitination of RIG-I, TBK1 and TRAF3, thereby blocking the expression of IFN-β [121]. Lpro not only deubiquitinates TBK1, it also cleaves its human version towards its C terminus, more specifically in the coiled-coil 2 (CC2) domain, at residues 693–694. However, contrary to what one might think, this interaction does not abolish or even decrease interferon production. Indeed, it has been shown that the cleavage product of TBK1 is able to perform the same functions as the full-length protein regarding interferon production. Thus, the N-terminal residue of TBK1 is sufficient for the phosphorylation of MAVS, as well as IRF3. This implies that the Lpro-mediated cleavage of TBK1 does not contribute to the viral strategy of the suppression of RLR signalling [50]. The role of this interaction therefore remains to be elucidated.

In addition to its action on the induction and production phases of the IFN pathway, Lpro also acts on the amplification phase of the antiviral response by interacting with RNase L. RNase L, or 2-5A-dependent ribonuclease, is an endoribonuclease activated by the oligoadenylate synthetase (OAS) whose expression is notably induced by IRF3 via their promoters, the IRF-binding domain [122]. RNase L is the enzymatic effector of a viral and cellular single-stranded mRNA degradation pathway, inducing successive cleavages and playing a prominent role in innate immunity. This cellular protein has also been shown to be capable of cleaving viral RNAs to directly eliminate the genome of single-stranded RNA viruses, such as EMCV [123]. Thus, the FMDV leader protein interacts with the N-terminal domain of porcine RNase L to prevent both the degradation of its mRNA and its genome. RNase L is a crucial mediator of innate immunity and other cell functions [61,124]. It is important to notice that in the experiment carried out by Sui et al., the interaction between Lpro and RNase L seems to be species-specific. Indeed, this interaction was demonstrated in pigs but could not be observed with endogenous forms in human, simian or canine cells [61]. It is therefore important to wonder what the situation is in other susceptible species, namely cow, sheep or goat, and whether these differences in interactions could explain the differences observed in the pathogenesis of the FMDV in these species [61]. RNA cleavage products by RNase L induce the formation of antiviral stress granules (avSGs). This is also the case for the Ras-GTPase-activating protein SH3 domain-binding proteins 1 and 2 (G3BP1 and G3BP2), scaffolding proteins that are essential for the stress granule formation mediated by the interferon-induced, double-stranded RNA-activated protein kinase (PKR). SGs are storage sites for mRNAs released from disassembled polysomes under stressful conditions, such as during viral infection. RNase L-, G3BP1- and G3BP1-induced avSGs allow the recruitment of the antiviral proteins RIG-I, PKR, OAS and additional RNase L. Through the interaction of G3BP1 with RIG-I, stress granules appear to signal RNase L-cleaved dsRNAs to RLRs, resulting in amplified IFN production. G3BP1 also participates in the amplification of type I IFN production by assisting RIG-I through its helicase activity [125]. To avoid such an interferon response amplification, FMDV Lpro, as well as ERAV Lpro cleaves human G3BP1 and G3BP2, preventing the formation of stress granules [60]. Interestingly, the proteases of these two viruses do not appear to cleave nor dephosphorylate PKR, and therefore they do not affect the integrated stress response (ISR) signalling cascade that leads to avSG formation.

### 4.2. 3C

The FMDV 3C proteinase is a chymotrypsin-like cysteine protease [126]. This protein is responsible for most of the cleavages that occur during viral polyprotein processing [127]. In addition to this role, this protein is also involved in counteracting the cellular response by inhibiting the transcription and translation of its host.

To this end, 3C notably acts on the induction phase of the interferon response. Thus, this viral protein limits the detection of exogenous RNA by RIG-I and MDA5 by decreasing the expression of exogenous swine LGP2 [62]. MDA5 is also a target of 3C, which reduces its level of translation and degrades it by protease action, preventing it from fulfilling its role in viral mRNA sensing [63].

The sensing of viral RNAs is also prevented by the FMDV thanks to the formation of membrane vesicles derived from the ER and/or the Golgi that binds replicase proteins. Indeed, these vesicles contain the neosynthesised viral RNA and protect it from helicases, such as RIG-I and MDA5 [128,129]. This remodelling of ER and Golgi membranes observed in simian cells results from a fragmentation of the early, middle and late Golgi compartments mediated by 3C proteolytic activity. This phenomenon also induces a blockage of the cell secretion system, which reduces the expression of the major histocompatibility complex (MHC) class I at the plasma membrane of infected cells. Furthermore, the consequences of these membrane rearrangements appear to be involved in facilitating the establishment of persistent FMDV infection [129].

The further induction of the IFN production pathway is also affected by swine NOD2- and RIP2 3C-mediated proteolytic cleavage [53,64]. NOD2 is able to detect both bacterial and viral recognition patterns. After binding to a viral ssRNA, NOD2 associates in a homo- or heterodimer with NOD1, allowing the recruitment of RIP2 via an interaction between their CARD domains. NOD2 contributes to the tyrosine phosphorylation of the rho guanine nucleotide exchange factor 2 (ARHGEF2), inducing the autophosphorylation of RIP2. After recruitment and autophosphorylation, RIP2 undergoes polyubiquitination, which is necessary for the recruitment of NEMO and MAP3K7, whose activation triggers the signalling cascade responsible for NF-kB activation. After recognising an RNA ligand, NOD2 is also able to interact with MAVS by association between the CARD domains. This interaction leads to the activation of IRF3 and thus to the initiation of the rest of the TLR pathway [32].

The 3C protein also limits viral RNA detection by inducing lysosomal degradation of nucleolar RNA helicase 2 (DDX21) in pigs [65]. This protein is involved in both the sensing of viral RNA and DNA initiating the signalling cascade of the IFN response and in the processing of ribosomal RNAs [130]. DDX21, which is already known to inhibit viral replication of influenza A virus (IAV) and human cytomegalovirus (HCMV), is also a negative regulator of the FMDV IRES-dependent translation [131,132]. The 3C-mediated degradation of DDX21 via the lysosomal pathway thus counteracts its direct action on IRES and dampens the induction of the IFN response.

The proteolytic activity of the 3C protein is also responsible for the cleavage of swine NEMO, at the Gln383 residue [66]. NEMO is composed of two coiled-coil domains, a leucine zipper, a conserved domain located at the N terminus that has been reported to bind to polyubiquitin chains and a zinc finger located at the C-terminus. While the coiled-coil domains and the leucine zipper are involved in most interactions between NEMO and activators of the IKK complex (NEMO–IKKα–IKKβ), it is interesting to note that this 3C-mediated cleavage targets a proline-rich region located just before the zinc finger domain [133]. This region, whose role is less known in NEMO, appears to be involved in MAP3K7 recruitment and in NF-kB activation. The 3C-mediated cleavage of swine NEMO thus prevents the recruitment of MAP3K7 and then the proteasome-mediated degradation of IκB required for NF-kB activation [134].

Finally, 3C protease affects the induction phase of the IFN pathway by degrading peroxiredoxin-6 (PRDX6) in swine. PRDX6 plays a major role in the inhibition of reactive oxygen species (ROS) production by affecting the formation of the complex between TRAF6 and evolutionarily conserved signalling intermediate in the toll pathway (ECSIT). ECSIT is an adaptor protein of the TLR signalling pathway, involved in the activation of NF-kappa-B and the promotion of MAP3K7 proteolytic activation. The TRAF6–ECSIT complex is required for the generation of ROS activating the toll-like receptor 4 (TLR4) responsible for the activation of the NF-κB and MAP3K pathways. To prevent the formation of this complex, PRDX6 interacts with the C-terminal domain of TRAF6, inducing its translocation into mitochondria. Therefore, PRDX6 reduces ROS production and thus TLR4-induced NF-κB activation [135]. However, upon infection, extracellular PRDX6 can directly activate the TLR4 receptor, inducing a signalling cascade responsible for NF-κB and AP-1-mediated proinflammatory cytokine production [136]. In addition, PRDX6 has been shown to inhibit FMDV replication via its acidic calcium-independent phospholipase A2 (aiPLA2) activity. This enzymatic activity is known to play an important role in phospholipid metabolism [137]. As phospholipid metabolism is linked to virus release mechanisms, it would be relevant to investigate whether PRDX6 inhibits the release of neoformed viruses. The FMDV 3C counteracts this antiviral response by cleaving swine PRDX6, as happens during SVV infection. This protein is also downregulated when pigs are infected with porcine circovirus 3 (PCV3) [67]. In contrast, in the case of classical swine fever virus (CSF) infection, PRDX6 was shown to be upregulated and to promote viral replication [138].

On the other hand, the FMDV 3C protease also targets the IFN production phase by blocking the transcription and translation steps. Indeed, 3C cleaves eIF4G (unmentioned host), but later than Lpro, targeting a different cleavage site [69]. In hamster cells, 3C cleaves another subunit of the eIF4 complex, namely the eukaryotic initiation factor 4A (eIF4A). eIF4A unwinds the secondary RNA structures at the 5′-UTR of mRNAs, which is necessary for the efficient binding of the small ribosomal subunit and subsequent search for the initiator codon. In the absence of integral eIF4A, mRNAs cannot be decondensed, they aggregate and result in the formation of stress granules [139]. The degradation of the eIF4 complex thus prevents the translation of host mRNAs to promote the translation of uncapped viral mRNAs [140].

Again, to inhibit the transcription of IFNs and other cytokines, 3C cleaves KH domain-containing, RNA-binding, signal transduction-associated protein 1, also known as Sam68. It is a positive regulator of mRNA stability, translation and nuclear export. It also binds to the poly(A) tail of mRNA to influence mRNA splice site selection and exon inclusion. The 3C-mediated cleavage of Sam68 induces the deletion of the C-terminal end containing the nuclear localization sequence (NLS), thus preventing its address to the nucleus. In addition, the approximately 50-kDa cleavage product, redistributed into the cytosol, interacts directly with the FMDV IRES and enhances viral RNA translation [72].

The mature ribosome (80S), a large ribonucleoprotein complex responsible for protein biosynthesis, is composed of a small ribosomal subunit (40S) and a large subunit (60S). The 60S subunit is composed of 49 ribosomal proteins (RPs), some of which are involved in the regulation of the NF-κB pathway. For instance, ribosomal protein S3 (RPS3) directly participates in the NF-κB complex by binding to the transcription factor p65 promoting the transcription of proinflammatory cytokines [141]. In contrast, ribosomal protein S27 (RPS27) blocks the phosphorylation of this same transcription factor, which reduces its DNA-binding capacity, and thus inhibits the transcription of cytokines. Ribosomal protein 60S L13 (RPL13) is known to interact with RIG-I and bind to the 3′-UTR region of NF-κB1 mRNA to promote its translation and increase NF-κB activity [142]. It has also been shown that RPL13, associated with the ATP-dependent RNA helicase DDX3, promotes the process of FMDV IRES-dependent translation required for its replication. However, this protein has an antiviral activity in response to FMDV infection as it can activate the promoters of NF-κB and IFN-β genes as well as the production and secretion of IFN-β and IL-6. To counteract this antiviral activity, FMDV 3C protease directly interacts with swine RPL13, inhibiting both gene and protein expression. While the resulting cleavage product no longer has antiviral activity, it is still capable of promoting the IRES-dependent translation of FMDV [71].

As for other picornaviruses, FMDV 3C has the ability to enter the nucleus of its host cell, thanks to a NLS located in the N-terminus of the 3CD protein precursor. Once in the nucleus, this protein can cleave many factors regulating cellular transcription. This is notably the case for histone H3, which is cleaved at its N-terminus in cattle [68]. Histone H3 is a central component of the nucleosome. Nucleosomes are responsible for the formation of chromatin by enveloping and compacting DNA to regulate DNA replication and transcription. Post-translational modifications of histones allow nucleosome remodelling. This results in variations in the level of chromatin compaction and thus changes in the accessibility of DNA to the cellular machinery. Among the various post-translational modifications that can affect histone proteins, modifications of their N-terminus are essential for chromatin decondensation. Indeed, nucleosome remodelling allows the release of binding sites of several transcription factors and thus the promotion of transcription [143]. Cleavage of H3 by 3C results in the removal of 20 N-terminal amino acids, preventing certain post-translational modifications from happening, thus shutting down host transcription.

The 3C protease is also able to cleave hamster heterogeneous nuclear ribonucleoprotein K (hnRNP K) at the Glu-364 residue during FMDV infection [70]. This premRNA binding protein is an IRES-transacting factor that enables ribosome recruitment and regulation of IRES translation. This protein is responsible for the translation shutdown and viral replication of the FMDV. Indeed, it contains KH2 and KH3 domains that bind to the IRES domains II, III and IV, preventing the binding of PTB, a positive regulatory factor of IRES translation. The 3C-mediated cleavage of hnRNP K results in two truncated forms of the protein that still possess some activity. Thus, the N-terminal cleavage product retains the KH2 domain but not the KH3, which confers a reduced inhibitory effect on IRES translation. In contrast, the C-terminal cleavage product positively regulates the FMDV replication, possibly by interacting with viral RNA other than the IRES. hnRNP M, a ribonucleoprotein known to have a structure and functions similar to hnRNP K, could bind RNA via its KH3 domain to antagonise their detection by RIG-I. It is therefore conceivable that the C-terminal cleavage product of hnRNP K acts in a similar way to inhibit the cellular response [144].

FMDV 3C not only limits the activation of the cellular response and the production of IFN. It further targets the JAK–STAT stage of the IFN response. This signalling pathway consists of type I IFN-sensing IFNARs. These are actually dimers of IFNAR1 and 2, with IFNAR2 having the highest affinity for IFNs. Upon binding of the IFN α or β, produced by their cell or surrounding cells, these membrane receptors trigger a phosphorylation cascade [42]. Thus, the tyrosine-protein kinase JAK1, associated with the IFNAR2 subunit, leads to the activation of TYK2, linked to the IFNAR1 subunit, by transphosphorylation on Tyr-1054 or Tyr-1055. Once activated, TYK2 can autophosphorylate its other tyrosine and then phosphorylate JAK1 in return [43]. Once activated, these two cytoplasmic kinases phosphorylate the tyrosine of the STAT family proteins, in particular, STAT1 and STAT2. The phosphorylated STAT1 and STAT2 form heterodimers that are translocated to the nucleus where they associate with the IFN regulatory factor 9 (IRF9) to form the transcription complex IFN-stimulated gene factor 3 (ISGF3). This complex in turn binds specifically to ISRE, present in most ISGs, to induce their expression and activate the antiviral response [145]. Similarly, STAT1 can also form homodimers that, when translocated to the nucleus, can enhance the transcription of many proinflammatory cytokines. 3C prevents this signalling cascade by blocking the nuclear translocation of human STAT1 and STAT2 to inhibit ISG expression. Specifically, 3C acts by degrading karyopherin α1 (KPNA1), the STAT1 NLS receptor, to prevent the translocation of STAT1–STAT2 or STAT1–STAT1 dimers to the nucleus and thus the activation of ISGs [75].

3C also targets interferon-induced proteins, such as PKR or G3BP1. PKR is a serine/threonine kinase that inhibits viral replication via the integrated stress response. This kinase is activated by the binding of the dsRNA or PKR activator protein (PACT), which leads to the phosphorylation of PKR. Once activated, it phosphorylates the alpha subunit of eukaryotic translation initiation factor 2 (eIF2α) to convert it into a global inhibitor of protein synthesis, stopping cellular and viral protein synthesis, while allowing the translation of ISR-specific mRNAs. PKR can also regulate the activation of signalling proteins and transcription factors, such as IRF3, NF-kB, AP-1 and STAT1 [146]. PKR also has another means of activating the NF-kB pathway in a manner independent of its kinase activity, namely interacting with the β-subunit of the IKK complex and binding to TRAF family proteins [147,148]. Many viruses have developed strategies to protect themselves from the antiviral effects of PKR. For example, the NS1 protein of IAV can bind to dsRNAs or even interact directly with PKR or prevent its activation [149]. Hepatitis C virus (HCV) IRES also inhibits PKR activation by competitively blocking its binding to its ligands [150]. Like these two viruses, the FMDV 3C counteracts the PKR-mediated antiviral response not via its proteolytic activity, but by inducing its lysosomal degradation in swine [74].

The ATP- and magnesium-dependent helicase G3BP1 is an antiviral factor capable, on the one hand, of enhancing the expression of RIG-I and MDA5 and, on the other hand, of significantly inhibiting the IRES-dependent translation of FMDV. This second activity requires the hyperphosphorylation of G3BP1 Ser149 mediated by casein kinase 2 (CK2). In order to combat this IRES shutdown-dependent translation, the FMDV has developed several strategies. The first aims to sequester the regulatory β-subunit of CK2 to prevent G3BP1 phosphorylation, while the second involves the proteolytic cleavage of porcine G3BP1 by 3C at the Glu284 residue [151]. Since the sequence surrounding the cleavage site is highly conserved between G3BP1 and G3BP2, it is quite possible that G3BP2 is also cleaved by 3C. This cleavage thus prevents the induction of antiviral sensor expression and the inhibition of IRES-dependent translation as well as the assembly of stress granules responsible for PKR and NF-κB activation [73].

### 4.3. 3A

FMDV 3A is a 153-amino-acid protein, the largest of the 3A proteins among the picornaviruses. This viral protein is particularly prone to point mutations in its C-terminal region that can affect the host specificity of the virus and its virulence. Thus, it was shown that a single mutation in the 3A sequence allowed the FMDV to infect guinea pigs, a nonnatural host for the virus [152]. Other studies have shown that deletions and substitutions in the 3A sequence can result in virus attenuation and reduced replication efficiency in cattle [153,154]. This protein is one of the least-conserved between different strains of the FMDV with only 37% invariant amino acids [155]. However, its N-terminus encoding a hydrophilic domain and a hydrophobic domain capable of binding to membranes is highly conserved [156]. This transmembrane domain is notably involved in associations with the membranes of Golgi and ER, a critical organelle for FMDV replication [157,158].

This hydrophobic region is necessary for the 3A inhibitory binding of the human proteins RIG-I, MDA5 and MAVS [76]. Indeed, RIG-I and MAVS bind to mitochondria-associated membranes (MAMs), an ER subdomain bridging mitochondria and the ER to establish synapses involved in innate immunity [159]. Targeting these membrane complexes with 3A thus prevents the efficient organisation of RLR signalling pathways, as is the case for the NS3 protein of HCV [160]. 3A also affects the expression of human RIG-I, MDA5 and MAVS by inhibiting their mRNA levels [76]. This results in a reduction in RNA-sensing activity and thus an inhibition of the induction phase of the IFN response mediated by the RLR pathway.

This protein also interacts with the swine-probable ATP-dependent RNA helicase DDX56 (DDX56), an enzyme involved in RNA metabolism and the negative regulation of virus-triggered IFN production [161]. 3A is able to hijack the regulatory activity of DDX56 to facilitate viral replication and avoid detection by the immune system. Thus, the cooperation between 3A and DDX56 induces a decrease in the phosphorylation level of IRF3, preventing it from associating with importin-5 (Imp5) which serves as an NLS receptor. Furthermore, via their interactions with the viral structural proteins VP0, VP1 and VP2, the 3A–DDX56 complex is able to inhibit the phosphorylation of other factors involved in IFN production, such as TBK1, IRF3, p65 and IκBa [79].

Furthermore, 3A has recently been shown to play a critical role in FMDV replication by interacting with swine annexin A1 (ANXA1) [78]. This member of the annexin family is known to be an important modulator of the immune response and particularly of the IFN pathway, since it is able to interact with MAVS, NEMO, IRF3 or TBK1 [162,163,164]. Regarding the action of this protein with regard to the FMDV, it has been shown that ANXA1-mediated IFNβ production significantly inhibits viral replication. The regulatory binding between 3A and ANXA1 enables the disruption of the ANXA1–TBK1 complex formation and thus limits the production of IFNβ associated with ANXA1 activity. The activity of 3A also focuses on the amplification phase of the IFN response by targeting G3BP1, following a different strategy than the ones adopted by 3C and Lpro. Indeed, 3A prevents G3BP1 antiviral activity through upregulating Leucine-rich repeat-containing protein 25 (LRRC25) in swine. LRRC25 is involved in the inhibition of the LRR pathway by inducing autophagic degradation of RIG-I. To this end, it directly interacts with RIG-I associated with the ubiquitin-like protein ISG15. The ubiquitination of RIG-I allows its interaction with the ubiquitin-binding protein p62, serving as a bridge between polyubiquitinated cargo and autophagosomes [165]. The upregulation of LRRC25 by 3A thus counteracts G3BP1-mediated IFN enhancement by helping RIG-I at sensing pathogenic RNA. This interaction between 3A and LRRC25, which was also demonstrated during EMCV and SVV infection, affects both the production of type I IFN and other proinflammatory cytokines [77].

### 4.4. 2B

Viroporin 2B is encoded by one of the most conserved regions of the FMDV genome, indicating its importance in the viral cycle [155]. This 154-amino-acid protein has two transmembrane domains involved in the rearrangement of host cell membranes. After 2B homodimerisation, its hydrophobic domains interact with the phospholipid bilayer to increase membrane permeability and facilitate the release of viral particles [166]. These membrane rearrangements are also involved in the formation of intracellular niches that prevent virus detection by the immune system. This protein is also involved in the disruption of cellular signalling pathways. Indeed, it mainly locates at the level of ER membranes where it interacts with 2C to block protein secretion, affecting the transport of MHC1 molecules [167]. 2B was also shown to interact with viral RNA sensors, such as swine LGP2. Thus, in porcine cells, 2B reduces the abundance of LGP2, while not affecting its transcription rate. This 2B-mediated degradation of LGP2 results from a yet unknown mechanism, independent of proteasome, lysosome and caspase pathways or the cleavage of eIF4G [62]. 2B also binds to human RIG-I and MDA5 resulting in a reduction in their viral detection ability [80]. Furthermore, these interactions result in the reduction of RIG-I and MDA5 expression in porcine cells [81]. Cyclophilin A (CypA) was recently shown to boost the RIG-I-mediated antiviral response by regulating the ubiquitination of RIG-I and MAVS [168]. Cyclophilin is also involved in reducing the abundance of viral proteins Lpro and 3A. 2B consequently targets swine CypA to prevent it from enhancing the IFN response [82]. 2B has also been reported to interact with other viral RNA sensors, such as swine NOD2 and DDX21 [64,65]. Given the wide range of viral sensors that this viral protein is capable of countering, it joins Lpro and 3C as frontline proteins against the IFN response initiation.

2B also acts on the signal transduction of the IFN pathway by targeting RIP2. The mechanism activated by 2B allowing the reduction of RIP2 abundance in pig cells still remains unknown and seems to be similar to that affecting RIG-I, LGP2 and NOD2. Nevertheless, this reduction in the RIP2 concentration limits the recruitment of MAP3K7 and NEMO and thus induces an inhibition of NF-kB activation mediated by the NLR pathway [53]. Moreover, viroporin 2B has been shown to interact with human TBK1 and IRF3 by limiting their phosphorylation. Unphosphorylated TBK1 decreases its ability to phosphorylate MAVS and recruit IRF3, while the inhibition of IRF3 phosphorylation prevents it from associating with Imp5, which is involved in its nuclear translocation. These indirect interactions caused by 2B are thus of major importance since it blocks an important part of the initiation phase of the TLR pathway [80].

2B also inhibits ISG transcription. Indeed, during the early phase of infection, it interacts with swine nucleoside diphosphate kinase A (NME1) to prevent it from enhancing the cellular tumour antigen p53 (p53) transcriptional activity [83]. NME1 is able to interact with the macrophage migration inhibitory factor (MIF) and E3 ubiquitin-protein ligase Mdm2 (MDM2), proteins with p53 downregulatory activity. The p53-mediated inhibition of IGS15, ISG20, IRF9 and RIG-I transcription thus allows the FMDV to initiate replication without being affected by IFN [169]. However, this interaction between 2B and NME1 has been shown to drop off during the late phase of infection, resulting in a basal level of p53-mediated transcription, which is necessary for viral replication to proceed [170].

### 4.5. VP1

Viral protein 1 is an extremely important structural protein in the FMDV viral cycle. It possesses an extended GH loop that includes an arginine-glycine-aspartic acid (RGD) motif, which mediates integrin binding [171]. Although adhesion to surface integrins is not the only viral entry route to the cell, VP1 allows the attachment and internalisation of the FMDV in the majority of natural infections [172]. This protein is among the first to be detected by the host cell. Indeed, VP1, is specifically recognised by human TLR2, through cooperation with TLR1 and TLR6 [36]. After adhesion of the capsid protein to TRL2, MyD88, a receptor-associated protein induces the recruitment of IRAK1 IRAK4, leading to the IRAK1 phosphorylation by IRAK4 and subsequent autophosphorylation and kinase activation. Once activated, IRAK1 recruits TRAF3 and TRAF6, responsible for its polyubiquitination. The ubiquitin chain thus formed is recognised by the ubiquitin-binding domain of NEMO and results in the formation of the IRAK1–MAP3K7–TRAF6–NEMO–IKKα–IKKβ complex. The formation of this complex then allows the activation of IKKα and IKKβ, which phosphorylate NF-kappa-B inhibitor alpha (IKBα), in order to induce its degradation as well as the nuclear translocation and the activation of NF-kB [173]. Similarly, VP1 is recognised by the human monocyte differentiation antigen CD14 (CD14), associated with the membrane receptor TLR4, leading to an enhancement of NF-kB early activation mediated by TLR2 [174]. The respiratory syncytial virus (RSV) and HCV also involve these receptors, initially known to be associated with the detection of bacterial agents, in the induction of proinflammatory cytokines in response to infection [175,176].

The role of FMDV structural proteins in the subversion of the immune system has been poorly documented in the past, but remains crucial. This is particularly the case for the VP1 protein, which is involved in the suppression of type 1 IFN response and particularly in the inhibition of the induction phase. On the one hand, this viral protein interacts specifically with the MAVS C-terminus (species used in this study not mentioned) [84]. This has the effect of blocking the TRAF3 binding site within MAVS, thus preventing its recruitment and thus the interferon-mediated response. On the other hand, VP1 is able to regulate the level of activation of the MAPK pathway [85]. Indeed, it can interact with swine ribosomal protein SA (RPSA), a protein involved in the assembly and the stability of the 40S ribosomal subunit as well as in the regulation of signalling transduction pathways. RPSA was shown to be a negative regulator of MAPK pathways by inducing the dephosphorylation of kinases responsible for the activation of the transcription factorAP-1, such as ERK1 and ERK2; c-Jun N-terminal kinase 1 (JNK1) and 2 (JNK2); and p38 mitogen-activated protein kinase (p38). The interaction between VP1 and RPSA represses the RPSA-mediated function and maintains the MAPK pathway activation that is necessary for viral replication. Other viruses also rely on MAPK pathway activation to carry out viral replication. This is notably the case of the Ebola virus (EBOV), which persistently infects and escapes from cells through the activation of the MAPK pathway and IAV, which takes advantage of airway inflammation induced by the MAPK pathway [177,178].

Conversely, VP1 can also inhibit TLR4-mediated activation of the MAPK/ERK pathway in swine macrophages by targeting tumour progression locus 2 (TPL2). Thus, VP1 promotes the K48-linked polyubiquitination of TPL2 that is a serine/threonine kinase required for the activation of the MAPK/ERK pathway. TPL2 thus ubiquitinated is recognised and degraded by the proteasome pathway and therefore is not considered to be effective for enhancing MAPK/ERK-mediated IFN production [87].

VP1 also influences the amplification phase of the IFN pathway by stimulating the activity of swine soluble resistance-related calcium binding protein (sorcin) via a yet unknown mechanism [86]. Sorcin is a calcium-binding protein involved in the suppression of type I interferon response. Indeed, this protein enhances the phosphorylation of the signal transducer and activator of transcription 3 (STAT3) on Tyr705 residue. STAT3 is a protein coupled to IL-6 receptors produced by NF-κB and activated by JAK1 to induce ISG expression. Once phosphorylated by sorcin, STAT3 could induce a negative feedback loop leading to a slowdown of NF-κB activation [179]. As NF-κB is involved in the regulation of many aspects of immune responses, a suppression of NF-κB signalling by engagement of the VP1 with sorcin, avoiding a cytokine storm, is among the elements that could contribute to the persistent infection of FMDV.

### 4.6. 2C

Protein 2C, composed of 318 amino acids, is one of the highly conserved molecules among FMDV proteins. It is also one of the most conserved proteins among Picornaviridae [180]. 2C has an amphipathic helix in its N-terminus allowing it to bind to cell membranes, including those of the ER and Golgi [181]. This membrane-binding ability makes 2C an important player in FMDV replication as it participates in the membrane-bound replication complex [167]. In addition, its association with 2B is involved in membrane rearrangements and alterations as well as the disruption of MHC1 molecule transport.

2C is also involved in the regulation of the host immune response during FMDV infection. Indeed, it interferes with the induction phase of the IFN response by inducing a decrease in the expression of swine NOD2, which limits the detection of viral ssRNA [64]. The mechanism involved in this viral antagonism remains poorly understood, but it has been shown that it does not depend on proteasomes, lysosomes, caspases, cleavage of eIF4G or the induction of apoptosis. Furthermore, among the truncated mutants tested, none was able to induce NOD2 reduction, indicating that multiple regions of 2C are involved in this phenomenon.

This viral protein also prevents IFN response initiation by inhibiting the DDX21 sensor. Like 2B, 2C does not interact directly with this helicase but induces its degradation via the caspase pathway rather than the lysosomal pathway as is the case for 3C [65].

2C also interacts with the swine E3 ubiquitin–protein ligase MARCHF7 [88]. The exact role of this interaction in FMDV replication is still unclear. However, MARCHF7 is known to enhance E2 activity of the ubiquitin-conjugating enzyme E2 K (HIP2), an enzyme capable of catalysing the synthesis of the polyubiquitin chain by elongating a monoubiquitinated substrate. HIP2 is involved in the suppression of apoptosis through the ubiquitination and degradation of p53 and may participate in the proteolytic processing of NF-kB by inducing its ubiquitination. It is thus possible to hypothesise that 2C promotes MARCHF7 activity to inhibit both autophagy and the NF-kB-mediated IFN response.

The swine protein RIP2 is also impacted by the antiviral activities of 2C. Indeed, 2C has the ability to induce a reduction in the level of polyadenylate-binding protein 1 (PABPC1), via a mechanism dependent on its amphipathic helix, probably by associating with proteinases. PABPC1 is involved in the regulation of processes of mRNA metabolism, such as premRNA splicing and mRNA stability, by binding the poly(A) tail of mRNA, including that of its own transcript. This 2C-mediated decrease in cellular PABPC1 concentration is thus responsible for the reduction of the RIP2 expression level and thus for a shutdown of the NLR pathway [53].

Furthermore, the action of 2C extends to the signalling phase of the IFN pathway by interacting with swine N-myc and STAT interacting (Nmi) and IFN-induced 35-kDa protein (IFP35) [89]. 2C is able to recruit these two proteins to the intracellular membrane by changing their subcellular distribution. Nmi is a signalling pathway regulator interacting with STAT family proteins in response to the detection of interleukin-2 (IL-2) or IFNγ. In particular, it allows the recruitment of STAT1 and STAT5 coactivators, inducing an increase in STAT-mediated transcription. The joint recruitment of Nmi and IFP35 results in the formation of the 2C–Nmi–IFP35 complex. This complex prevents the proteasome-mediated degradation of IFI35 and correlates with IFI35 dephosphorylation, inducing the inhibition of the nuclear translocation, activation and transcription of the NF-kappa-B subunit p65.

### 4.7. VP3

Viral protein 3 is a structural protein that has been implicated in the pathogenicity of the FMDV. It has been shown that its amino acid at position 56, which can be a histidine, arginine or cysteine, influences pathogenesis in cattle and swine [182,183,184]. Indeed, the presence of an arginine in this region participates in the binding to cellular HS, a coreceptor used by some strains, by increasing the positive charge of the viral surface. The binding with cellular HS, negatively charged, is thus reinforced, which may induce a sequestration of the virus at the level of these receptors and limit the propagation of the virus, explaining in vivo findings [185].

Similarly to VP1, VP3 is recognised by TLR2 and by CD14 as being associated with TLR4, thus triggering the signalling cascade of the TLR pathway, responsible for NF-kB activation [36]. VP3 can also induce TLR4 overexpression by interacting with the human ras-related protein Rab-7b (Rab7b). To do so, VP3 drastically reduces the expression of this protein responsible for the lysosomal degradation of TLR4. This results in an enhancement of the FMDV-elicited inflammatory response mediated by TLR4 [37]. However, as well as other structural proteins, VP3 participates in the counteracting of host antiviral activity by diverting cellular chaperone proteins. In fact, the assembly of the FMDV capsid requires the intervention of cellular chaperone proteins. Thus, it has been shown that chaperones, such as heat shock protein HSP 90 (HSP90), initially involved in the immune response, are necessary for the assembly of the FMDV capsid [186]. One of the roles of these chaperones is to ensure the conformation of the pathogen recognition receptors. The occupation of these chaperone proteins by viral structural proteins therefore prevents the correct folding of these receptors and thus the detection of infection.

VP3 also acts by specifically targeting one of the most important elements in signal transduction, namely MAVS. Indeed, the C-terminal domain of VP3 has been shown to interact with the transmembrane domain of human and swine MAVS (amino acids 470–540) resulting in the disruption of MAVS mitochondrial localisation. [187] This interaction leads to the inhibition of MAVS expression by interfering with its mRNA synthesis. The decrease in the MAVS level thus constitutes an important brake in the signal transduction of the IFN pathway. It was thus observed that it notably led to a block of the phosphorylation and dimerization of IRF3 required for the production of IFN via the RLR pathway [90].

In addition, VP3 antagonises the host antiviral response by preventing signal amplification through the JAK/STAT pathway. Indeed, it is able to bind to JAK1 and JAK2 in order to induce the lysosome-mediated degradation of JAK1 and prevent the interaction between JAK1 and STAT1. This interaction is required for the phosphorylation and dimerization of STAT1 and thus results in the inhibition of ISG transcription. Surprisingly, it has also been shown that JAK1 and JAK2 can increase the expression of VP3, increasing the efficiency of its antiviral action. The mechanism responsible for this JAK-mediated overexpression of VP3 is not yet fully understood [91].

### 4.8. VP0

The cleavage of the intermediate protein VP0 into VP4 and VP2 is the result of a mechanism that is not yet fully understood. This event occurs in the late stages of the viral replication cycle, just before genomic RNA encapsidation [188].

The VP0 preprotein is able to bind to swine poly(rC)-binding protein 2 (PCBP2) to prevent the establishment of the IFN response. PCBP2 is a single-stranded RNA and DNA-binding protein involved in the negative regulation of MAVS-mediated antiviral responses. Lacking a catalytic domain capable of directly degrading this protein, PCBP2 recruits the HECT domain-containing E3 ligase AIP4 to polyubiquitinate MAVS and to induce its degradation via the apoptotic pathway [189]. The interaction between VP0 and PCBP2 thus enhances the antiMAVS activity of the host cell [92]. It has also been shown that, upon poliovirus infection (PV), PCBP2 can stimulate the IRES-mediated translation and play a role in the initiation of viral RNA replication by interacting with the 3CD protein [190].

Viral protein 4 is the smallest of the FMDV capsid proteins and is located on the inner surface of the capsid. VP4 plays a key role in the pentamer assembly by modulating both antigenic status and receptor binding. It is also secondarily involved in the silencing of the immune response. To this end, VP4 induces the degradation of the swine transcriptional activator NME1 through the macroautophagy pathway [83]. This indirect interaction thus promotes the association between p53 and its repressors MDM2 and MIF to decrease antiviral activity in host cells and promote viral replication. This downregulation of NME1 is also thought to activate the MAPK pathway and autophagy mechanisms that may promote FMDV replication [191].

### 4.9. 3B

3B, also known as VPg, is one of the viral proteins that is involved in initiating viral RNA synthesis. Unlike most other picornaviruses, the FMDV has not one but three similar copies of 3B, namely 3B1, 3B2 and 3B3. These three copies of 23 to 24 amino acids are very similar but not identical [154]. The first step of FMDV genome replication is the attachment of one copy of 3B to the RNA via its highly conserved tyrosine residue in the 3rd position. The 3D polymerase then catalyses the uridylylation of this amino acid to allow synthesis of the viral RNA. All three isoforms of 3B can serve as substrates for 3D. However, 3B3 has been shown to be the most efficient, to the extent that a virus deleted from 3B3 results in the production of noninfectious RNA [192]. Viruses lacking 3B1 and 3B2 can induce infectious RNA but with a low level of synthesis, resulting in reduced viral replication [193]. While all previously known strains had three copies of 3B, the O/SKR/01/2014 strain identified in South Korea contained a mutation deleting copy 3B1. Interestingly, it was shown that this strain appeared to be relatively nonvirulent in pigs, indicating that the presence of different copies of 3B could be a factor in determining the pathogenicity of the FMDV [194].

Few interactions between 3B and the immune response have yet been identified. However, this protein participates in the inhibition of the IFN response induction by interacting with swine RIG-I [93]. Indeed, 3B interacts with the RIG-I CARD domain to prevent its interaction with TRIM25. This E3 ubiquitin ligase cannot therefore induce the ubiquitination of RIG-I, which is necessary for the formation of the MAVS–RIG-I complex. It was also shown that 3B could bind to the RIG-I DEAD helicase domain, implicated in the IFN response in an ubiquitin-independent manner, to block its interaction with RIPLET E3 ligase [195].

### 4.10. 3D

3D is the last protein encoded by the FMDV genome. This region is highly conserved among different viral strains. 3D is an RNA-dependent RNA polymerase that becomes active once processing of the 3CD preprotein is complete [196]. This polymerase locates mainly near other nonstructural proteins in the ER and Golgi membranes. It is the catalytic component required for viral RNA replication and plays an important role in the virus life cycle. As with 3B, the involvement of the 3D protein in combating the antiviral response is considered secondary to its role in viral replication. Indeed, only two interactions have been demonstrated to date between this protein and the immune system of its hosts. Thus, like PV 3D, FMDV 3D is able to bind specifically with mouse Sam68 [197]. This binding, permitted by the charge complementarity between the binding surfaces of the two proteins, prevents Sam68 translocation to the nucleus [94]. Furthermore, 3D has been shown to interact with swine ATP-dependent RNA helicase DDX1 (DDX1) [95]. This protein is involved in both the inhibition of FMDV replication and the production of type I IFN. The biological significance of the 3D/DDX1 interaction remains unresolved to date.

## 5. Discussion and Concluding Remarks

As shown by the large number of studies cited in this review, the FMDV dedicates almost its entire small genome to fighting the immune system and especially the type I interferon response.

While some cellular proteins are targeted by a single viral protein, other targets are shared by several FMDV proteins. This is notably the case for the main proteins responsible for sensing viral RNAs and the associated signalling proteins involved in the RLR and NLR pathways, which are particularly targeted. As for the sensors, they have been described as interacting with various FMDV nonstructural proteins. Thus, RIG-I is targeted by Lpro, 2B, 3A and 3B; MDA5 is affected by Lpro, 3B, 3A and 3C; LGP2 interacts with Lpro, 2B and 3C, while NOD2 is focused by 2B, 2C and 3C. Regarding signalling proteins, MAVS and RIP2 are targeted by Lpro, 3A, VP1 and VP3 and Lpro, 2B and 3C, respectively. In the framework of these interactions, the FMDV viral proteins involve various mechanisms, such as proteolytic cleavage, inhibitory binding, inhibition of mRNA transcription levels, deubiquitination and other unknown mechanisms. The diversity of viral proteins responsible for the inhibition of the induction phase of the IFN pathway, associated with the diversity of targeted cellular proteins, as well as the range of mechanisms used, allows the FMDV to bypass the immune response in an even more efficient way and, above all, to regulate the equilibrium with its host in an extremely fine manner. The interactions between the FMDV and the proteins located in the upstream phase of the induction of the IFN pathway seem all the more important as these proteins are also major targets for many other viruses, including the picornaviruses [198]. Thus, the critical role of MDA5 in the recognition of viral RNAs is also limited by the enterovirus 71 (EV71) via 2A and 3D polymerase [199,200]. The same happens with the coxsackievirus B3 [201]. The 3C of the coxsackievirus A6 and A16, as well as the enterovirus D68, have also been described as being able to perform inhibition-binding on MDA5 [202]. In addition, 2C and VP2 of EMCV have been shown to interact with MDA5, while 2A of PV also appears to be responsible for the degradation of this sensor [203,204]. A significant proportion of the above proteins are also reported to interact with other players involved in the IFN response induction phase, in particular RIG-I and MAVS. In the light of the current literature, some picornaviruses seem to focus more on these last two targets rather than on MDA5. This is notably the case of HRV-1A, which targets MAVS via 2A and 3C, hepatitis A virus (HAV), which also targets MAVS via 2B, and precursor 3ABC [205,206]. This is also the case for SVV, which blocks the action of RIG-I via 2C and 3C, a protein also capable of cleaving MAVS and other proteins situated further downstream of the IFN pathway [121,207,208]. Although the means used differ between viruses, these targets are nonetheless conserved, proving their importance in setting up the immune response.

While most of the escape strategies used by the FMDV are now well-characterised, others still require further research. In particular, it is essential to understand how and why the observations made differ from one cell line to another or from one species to another.

More than half of the interactions described in this review were demonstrated using the overexpression of proteins from species not susceptible to the FMDV, human in most cases (full list is available in Table 1). It is therefore interesting to ask whether these interactions exist in the natural hosts of the virus. Only a few articles mention having tested the interaction in other species. This is notably the case for the work of Sui et al. on the interaction between Lpro and RNase L [61]. This has been demonstrated in a porcine cell model but not in human, simian and canine models. The lack of conservation of this interaction from one species to another encourages us to take a step back from the interactions demonstrated with models that are not always adapted to the study of this virus. However, this questioning must be balanced by the conservation of other interactions between species. For instance, this is the case of the interaction between Lpro and LGP2, which has been demonstrated in swine and human models [49]. The same is true for the interaction between VP3 and MAVS, which is not species-specific as it was detected in both swine and human in vitro models by Ekanayaka et al. [187].

Questioning the host specificity of these interactions could contribute to the development of new hypotheses to explain the differential persistence of the FMDV. The mechanisms of establishment, maintenance and resolution of FMDV persistence remain indeed unresolved to date, but both in vitro and in vivo work indicates the coevolution of the FMDV and host cells during persistence [22,24,25]. It thus seems relevant to investigate whether virus–host interactions present in species for which persistence is described, such as cattle and small ruminants, are conserved or not in species, such as swine, for which viral persistence has not been described. The same is true for interactions that could be found in species that do not show persistence but not in species where persistence is described.

Moreover, it also seems important to ask in which infectious context these interactions can be identified. Indeed, according to the work of Hägglund et al. and Pfaff et al., which made it possible to characterise the signatures of a bovine epithelial cell model of FMDV infection, the host responses are not identical depending on whether the infection is acute or persistent [209,210]. It was found that in this model, acute infection is characterised by a high transcription of ISGs leading to high antiviral activity, whereas persistent infection is accompanied by a long-lasting but limited innate antiviral response that is ineffective at clearing the virus. Among the 73 ISGs whose expression is modified during persistent FMDV infection, 10 are different from those overexpressed during acute infection, potentially implying different interactions between the virus and its host depending on the context of the infection [210].

In order to better understand the persistence of the FMDV, other cellular mechanisms also deserve to be studied, namely autophagy and apoptosis. Autophagy is a highly conserved process in all eukaryotes. It is constitutively involved in the partial degradation of unnecessary cytosolic constituents or organelles via their engulfment within double-membraned vesicles named autophagosomes, which fuse with lysosomes to form autolysosomes where degradation occurs. Its main form, macroautophagy, can be upregulated upon virus detection by pathogen receptors to initiate the degradation of certain viruses, giving it a prominent role in the antiviral response [211,212].

One of the major pieces of evidence of the importance of autophagy regarding the antiviral response is probably the multitude of evasion and subversion mechanisms implemented by viruses. The FMDV is not an exception to this rule, since several of its proteins have been shown to be involved in interactions with autophagy proteins. In particular, DnaJ homolog subfamily A member 3 (DNAJA3) has been shown to interact with VP1, leading to its degradation by the lysosomal pathway [213]. This weakens the inhibition of the IFN response mediated by VP1. DNAJA3, notably through its interactions with Beclin-1 and autophagy-related protein LC3 (LC3), allows the induction of macroautophagy and chaperone-mediated autophagy in order to limit the development of the infection. The IFN response-boosting role of autophagy is also highlighted by the upregulation of NF-κB and IRF3 activity by ATG5 and ATG12 during FMDV infection, as these autophagy proteins could also interact with VP1 [24]. Similarly, the interaction between 2C and MARCHF7 inhibits both autophagy and the NF-kB-mediated IFN response. While autophagy might be thought to be an antiFMDV action mechanism, there are IPP that greatly qualify this statement. For example, it has been shown that VP2 activates the cellular EIF2S1–ATF4 pathway and promotes autophagy via heat shock protein beta-1 (HSPB1) to facilitate viral replication [214]. Similarly, the interactions described between NME1 and the viral proteins 2B and VP4, in addition to their inhibitory effect on the IFN pathway, also appear to have a function in amplifying autophagy, allowing a better replication of the virus. The 2C protein seems to play an important role in the autophagy regulation by the FMDV. Indeed, its interaction with vimentin has been shown to be essential for FMDV replication. In addition, it is able to interact with Beclin-1 to prevent the lysosomes from fusing into autophagosomes [215]. This phenomenon, which has already been identified during infection by other RNA viruses, considerably promotes viral replication through a mechanism that is not yet understood [191]. Interestingly, some autophagy-mediated mechanisms of the facilitation of FMDV replication have been demonstrated in relevant models for the study of persistent infection [215,216]. This could suggest that under certain conditions, autophagy enables the nonlytic release of the virion from the cells. Autophagy could thus be involved in the establishment and/or maintenance of the virus–host balance that leads to persistent infections. The importance of autophagy should also be emphasised in view of its close interplay with the IFN pathway. Indeed, it has been shown that autophagy can regulate the IFN response, while IFNs and ISGs derived from this pathway can also act on autophagy [217,218]. Likewise, the interactions between the virus and the type I IFN response and autophagy are closely linked to apoptosis. Indeed, this programmed cell death process is exploited by the FMDV to eliminate some components of the IFN response. This is particularly the case for MAVS whose degradation is mediated by the VP0–PCBP2 interaction [189]. In the case of DDX21, it is interesting to notice that its degradation can be due to apoptosis phenomena when mediated by 2B or autophagy-related mechanisms when mediated by 2C or 3C [65]. In contrast, many FMDV–host interactions aim to inhibit the establishment of apoptosis in cells by targeting the proapoptotic factor p53. This is, for example, the case of the 2B–NME1 interaction, which prevents the enhancement of p53 activity by NME1 [83]. The same is true for the interactions of VP4 and 2B with NME1. Apoptosis also plays an important position in the antiviral response as evidenced by the interaction between proapoptotic serine/threonine-protein kinase 3 (STK3) and VP1, which results in viral replication inhibition [219]. Although autophagy is more involved in the early response to double-stranded RNA detection, apoptosis takes over at prolonged stages of stress. RNase L is one of the key factors in this transition by activating the caspase-dependent proteolytic cleavage of Beclin-1 to terminate autophagy and promote apoptosis. RNase L is therefore a key player in the regulation of the antiviral response, explaining why it is targeted by Lpro.

The result of these intertwined signalling pathways is an extremely fine regulation of the antiviral response, but which can be hijacked at many positions by the virus. As the interactions described between the FMDV and the cellular response pathways sometimes have contradictory effects, it would be interesting to investigate, in addition to the models used, the chronological occurrence of these interactions. Some of them may only take place at the beginning of the infection, while others could participate in the maintenance of a virus–host balance in the longer term and potentially in the persistence of the FMDV. Studying the protein interactions between the FMDV and its different hosts, combined with transcriptomic analyses in relevant cell models from species of interest, could greatly contribute to the improvement of knowledge on the differential persistence of this virus. This could open up new therapeutic avenues for the control of safe carriers.

## Figures and Tables

**Table 1:** Protein–protein interactions described between FMDV 3D polymerase and type I IFN response.

Viral Protein			Cellular Target			Strategy	Reference	
Name	Nature	Viral Strain	Name	Impacted Biological Mechanism	Protein Origin	Action Mechanism	Title	Author
**Lpro**	NS	O1K	**LGP2**	Signal transmitting	Human	Cleavage	Innate immune sensor LGP2 is cleaved by the Leader protease of foot-and-mouth disease virus	Rodriguez Pulido (2018) [49]
**Lpro**	NS	A12	**MAVS**	Signal transmitting	Swine	N.D	Dissecting distinct proteolytic activities of FMDV Lpro implicates cleavage and degradation of RLR signaling proteins, not its deISGylase/DUB activity, in type I interferon suppression	Visser (2020) [50]
**Lpro**	NS	O1K	**MDA5**	Signal transmitting	Human	Cleavage	MDA5 Cleavage by the Leader Protease of Foot-and-Mouth Disease Virus Reveals Its Pleiotropic Effect against the Host Antiviral Response	Pulido (2020) [51]
**Lpro**	NS	O/ES/2001	**RIG-I**	Signal transmitting	Human	Cleavage	The leader proteinase of foot-and-mouth disease virus negatively regulates the type I interferon pathway by acting as a viral deubiquitinase	Wang (2011) [52]
**Lpro**	NS	O/BY/CHA/2010	**RIP2**	Signal transmitting	Swine	Cleavage	Foot-and-Mouth Disease Virus Inhibits RIP2 Protein Expression to Promote Viral Replication	Liu (2021) [53]
**Lpro**	NS	O/ES/2001	**TRAF3**	Signal transmitting	Human	Deubiquitination	The leader proteinase of foot-and-mouth disease virus negatively regulates the type I interferon pathway by acting as a viral deubiquitinase	Wang (2011) [52]
**Lpro**	NS	O/ES/2001	**TRAF6**	Signal transmitting	Human	Deubiquitination	The leader proteinase of foot-and-mouth disease virus negatively regulates the type I interferon pathway by acting as a viral deubiquitinase	Wang (2011) [52]
**Lpro**	NS	O/ES/2001	**IRF3**	Transcription	Swine	Cleavage	Foot-and-mouth disease virus leader proteinase inhibits dsRNA-induced type I interferon transcription by decreasing interferon regulatory factor 3/7 in protein levels	Wang (2010) [54]
**Lpro**	NS	O/ES/2001	**IRF7**	Transcription	Swine	Cleavage	Foot-and-mouth disease virus leader proteinase inhibits dsRNA-induced type I interferon transcription by decreasing interferon regulatory factor 3/7 in protein levels	Wang (2010) [54]
**Lpro**	NS	A12	**p65**	Transcription	Goat	Cleavage	Degradation of Nuclear Factor Kappa B during Foot-and-Mouth Disease Virus Infection	De Los Santos (2007) [55]
**Lpro**	NS	A12	**ADNP**	Transcription	Human	Regulatory binding	Interaction between FMDV L pro and transcription factor ADNP is required for optimal viral replication	Medina (2017) [56]
**Lpro**	NS	C-S8	**eIF3a**	Translation	Human	Cleavage	Foot-and-mouth disease virus infection induces proteolytic cleavage of PTB, eIF3a,b, and PABP RNA-binding proteins	Rodriguez Pulido (2007) [57]
**Lpro**	NS	C-S8	**eIF3b**	Translation	Human	Cleavage	Foot-and-mouth disease virus infection induces proteolytic cleavage of PTB, eIF3a,b, and PABP RNA-binding proteins	Rodriguez Pulido (2007) [57]
**Lpro**	NS	O6	**eIF4GI**	Translation	Human	Cleavage	Leader Protein of Foot-and-Mouth Disease Virus Is Required for Cleavage of the p220 Component of the Cap-BindingProteinComplex	Devaney (1988) [58]
**Lpro**	NS	O6	**eIF4GII**	Translation	Human	Cleavage	Leader Protein of Foot-and-Mouth Disease Virus Is Required for Cleavage of the p220 Component of the Cap-BindingProteinComplex	Devaney (1988) [58]
**Lpro**	NS	C-S8	**Gemin-5**	Translation	Human	Cleavage	Gemin5 proteolysis reveals a novel motif to identify L protease targets	Pineiro (2012) [59]
**Lpro**	NS	C-S8	**PABP**	Translation	Human	Cleavage	Foot-and-mouth disease virus infection induces proteolytic cleavage of PTB, eIF3a,b, and PABP RNA-binding proteins	Rodriguez Pulido (2007) [57]
**Lpro**	NS	C-S8	**PTB**	Translation	Human	Cleavage	Foot-and-mouth disease virus infection induces proteolytic cleavage of PTB, eIF3a,b, and PABP RNA-binding proteins	Rodriguez Pulido (2007) [57]
**Lpro**	NS	A12	**G3BP1**	Response amplification	Human	Cleavage	Foot-and-Mouth Disease Virus Leader Protease Cleaves G3BP1 and G3BP2 and Inhibits Stress Granule Formation	Visser (2019) [60]
**Lpro**	NS	A12	**G3BP2**	Response amplification	Human	Cleavage	Foot-and-Mouth Disease Virus Leader Protease Cleaves G3BP1 and G3BP2 and Inhibits Stress Granule Formation	Visser (2019) [60]
**Lpro**	NS	O/Tibet/CHA/99	**RNase L**	Response amplification	Swine	Regulatory binding	Inhibition of Antiviral Innate Immunity by Foot-and-Mouth Disease Virus Lpro through Interaction with N-terminal Domain of Swine RNase L	Sui (2021) [61]
**Lpro**	NS	O/ES/2001	**TBK1**	N.D	Human	Deubiquitination and Cleavage	The leader proteinase of foot-and-mouth disease virus negatively regulates the type I interferon pathway by acting as a viral deubiquitinase	Wang (2011) [52]
**3C**	NS	O/BY/CHA/2010	**LGP2**	Viral sensing	Swine	Inhibition of protein expression	Foot-and-mouth disease virus infection inhibits LGP2 protein expression to exaggerate inflammatory response and promote viral replication	Zhu (2017) [62]
**3C**	NS	O/SKR/2000	**MDA5**	Viral sensing	Swine	Inhibition of protein expression	Foot-and-Mouth Disease Virus Evades Innate Immune Response by 3C-Targeting of MDA5	Kim (2021) [63]
**3C**	NS	O/BY/CHA/2010	**NOD2**	Viral sensing	Swine	Cleavage	Foot-and-Mouth Disease Virus Antagonizes NOD2-Mediated Antiviral Effects by Inhibiting NOD2 Protein Expression	Liu (2019) [64]
**3C**	NS	O/BY/CHA/2010	**DDX21**	Viral sensing and Signal transmitting	Swine	Lysosomal degradation	DDX21, a Host Restriction Factor of FMDV IRES-Dependent Translation and Replication	Abdullah (2021) [65]
**3C**	NS	O/ES/2001	**NEMO**	Signal transmitting	Swine	Cleavage	Foot-and-Mouth Disease Virus 3C Protease Cleaves NEMO To Impair Innate Immune Signaling	Wang (2012) [66]
**3C**	NS	O/BY/CHA/2010	**RIP2**	Signal transmitting	Swine	Cleavage	Foot-and-Mouth Disease Virus Inhibits RIP2 Protein Expression to Promote Viral Replication	Liu (2021) [53]
**3C**	NS	O/BY/CHA/2010	**PRDX6**	Signal transmitting	Swine	Cleavage	Porcine Picornavirus 3C Protease Degrades PRDX6 to Impair PRDX6-mediated Antiviral Function	Wang (2021) [67]
**3C**	NS	O1K	**H3**	Transcription	Cattle	Cleavage	Foot-and-mouth disease virus protease 3C induces specific proteolytic cleavage of host cell histone H3	Falk (1990) [68]
**3C**	NS	CS8	**eIF4A**	Translation	Hamster	Cleavage	Foot-and-Mouth Disease Virus 3C Protease Induces Cleavage of Translation Initiation Factors eIF4A and eIF4G within Infected Cells	Belsham (2000) [69]
**3C**	NS	CS8	**eIF4G**	Translation	Hamster	Cleavage	Foot-and-Mouth Disease Virus 3C Protease Induces Cleavage of Translation Initiation Factors eIF4A and eIF4G within Infected Cells	Belsham (2000) [69]
**3C**	NS	O/YS/CHA/05	**hnRNP K**	Translation	Hamster	Cleavage	hnRNP K Is a Novel Internal Ribosomal Entry Site-Transacting Factor That Negatively Regulates Foot-and-Mouth Disease Virus Translation and Replication and Is Antagonized by Viral 3C Protease	Liu (2020) [70]
**3C**	NS	O/BY/CHA/2010	**RPL13**	Translation	Swine	Cleavage	Ribosomal Protein L13 Participates in Innate Immune Response Induced by Foot-and-Mouth Disease Virus	Guan (2021) [71]
**3C**	NS	A24/Cruzeiro/Brazil 1955	**Sam68**	Translation	Human	Cleavage	The nuclear protein Sam68 is cleaved by the FMDV 3C protease redistributing Sam68 to the cytoplasm during FMDV infection of host cells	Lawrence (2012) [72]
**3C**	NS	O/ES/2001	**G3BP1**	Response amplification	Swine	Cleavage	Foot-and-Mouth Disease Virus Counteracts on Internal Ribosome Entry Site Suppression by G3BP1 and Inhibits G3BP1-Mediated Stress Granule Assembly via Post-Translational Mechanisms	Ye (2018) [73]
**3C**	vS	O/BY/CHA/2010	**PKR**	Response amplification	Swine	Lysosomal degradation	Foot-and-mouth disease virus induces lysosomal degradation of host protein kinase PKR by 3C proteinase to facilitate virus replication	Li (2017) [74]
**3C**	NS	O/Tibet/CHA/99	**STAT1**	Response amplification	Human	Prevention of nuclear translocation	3Cpro of Foot-and-Mouth Disease Virus Antagonizes the Interferon Signaling Pathway by Blocking STAT1/STAT2 Nuclear Translocation	Du (2014) [75]
**3C**	NS	O/Tibet/CHA/99	**STAT2**	Response amplification	Human	Prevention of nuclear translocation	3Cpro of Foot-and-Mouth Disease Virus Antagonizes the Interferon Signaling Pathway by Blocking STAT1/STAT2 Nuclear Translocation	Du (2014) [75]
**3A**	NS	O/Tibet/CHA/99	**RIG-I**	Viral sensing	Human	Regulatory binding and reduction of RNA levels	Foot-and-mouth disease virus non-structural protein 3A inhibits the interferon-β signaling pathway	Li (2016) [76]
**3A**	NS	type-O	**LRRC25**	Signal transmitting	Swine	Protein upregulation	Foot-and-Mouth Disease Virus 3A Protein Causes Upregulation of Autophagy-Related Protein LRRC25 To Inhibit the G3BP1-Mediated RIG-like Helicase-Signaling Pathway	Yang (2020) [77]
**3A**	NS	O/Tibet/CHA/99	**MAVS**	Signal transmitting	Human	Regulatory binding and reduction of RNA levels	Foot-and-mouth disease virus non-structural protein 3A inhibits the interferon-β signaling pathway	Li (2016) [76]
**3A**	NS	O/Tibet/CHA/99	**MDA5**	Signal transmitting	Human	Regulatory binding and reduction of RNA levels	Foot-and-mouth disease virus non-structural protein 3A inhibits the interferon-β signaling pathway	Li (2016) [76]
**3A**	NS	O/BY/CHA/2010	**ANXA1**	Signal transmitting	Swine	Regulatory binding	FMDV 3A Antagonizes the Effect of ANXA1 to Positively Modulate Viral Replication	Ma (2022) [78]
**3A**	NS	O/BY/CHA/2010	**DDX56**	Translation	Swine	Regulatory binding	DDX56 cooperates with FMDV 3A to enhance FMDV replication by inhibiting the phosphorylation of IRF3	Fu (2019) [79]
**2B**	NS	O/BY/CHA/2010	**DDX21**	Viral sensing	Swine	N.D	DDX21, a Host Restriction Factor of FMDV IRES-Dependent Translation and Replication	Abdullah (2021) [65]
**2B**	NS	O/Tibet/CHA/2010	**LGP2**	Viral sensing	Swine	N.D	Foot-and-mouth disease virus infection inhibits LGP2 protein expression to exaggerate inflammatory response and promote viral replication	Zhu (2017) [62]
**2B**	NS	O/MYA/01/1998	**MDA5**	Viral sensing	Human	Regulatory binding	Foot-and-mouth disease virus non-structural protein 2B negatively regulates the RLR-mediated IFN-β induction	Li (2018) [80]
**2B**	NS	O/BY/CHA/2010	**NOD2**	Viral sensing	Swine	N.D	Foot-and-Mouth Disease Virus Antagonizes NOD2-Mediated Antiviral Effects by Inhibiting NOD2 Protein Expression	Liu (2019) [64]
**2B**	NS	O/BY/CHA/2010	**RIG-I**	Viral sensing	Swine	Inhibition of protein expression	Foot-and-Mouth Disease Virus Viroporin 2B Antagonizes RIG-I-Mediated Antiviral Effects by Inhibition of Its Protein Expression	Zhu (2016) [81]
**2B**	NS	O/MYA/01/1998	**RIG-I**	Viral sensing	Human	Regulatory binding	Foot-and-mouth disease virus non-structural protein 2B negatively regulates the RLR-mediated IFN-β induction	Li (2018) [80]
**2B**	NS	O/BY/CHA/2010	**Cyclophilin A**	Signal transmitting	Swine	N.D	Foot-and-mouth disease virus nonstructural protein 2B interacts with cyclophilin A, modulating virus replication	Liu (2018) [82]
**2B**	NS	O/BY/CHA/2010	**RIP2**	Signal transmitting	Swine	N.D	Foot-and-Mouth Disease Virus Inhibits RIP2 Protein Expression to Promote Viral Replication	Liu (2021) [53]
**2B**	NS	O/BY/CHA/2010	**NME1**	Transcription	Swine	N.D	Foot-and-mouth disease virus induces lysosomal degradation of NME1 to impair p53-regulated interferon-inducible antiviral genes expression	Feng (2018) [83]
**VP1**	VP	O/Taiwan/97	**TLR2**	Viral sensing	Human	Regulatory binding	Capsid proteins of foot-and-mouth disease virus interact with TLR2 and T CD14 to induce cytokine production	Lin (2020) [36]
**VP1**	VP	O/Taiwan/97	**CD14**	Signal transmitting	Human	Regulatory binding	Capsid proteins of foot-and-mouth disease virus interact with TLR2 and T CD14 to induce cytokine production	Lin (2020) [36]
**VP1**	VP	O1/Manisa/Turkey/69	**MAVS**	Signal transmitting	N.D	Regulatory binding	Foot-and-mouth disease virus VP1 target the MAVS to inhibit type-I interferon signaling and VP1 E83K mutation results in virus attenuation	Ekanayaka (2020) [84]
**VP1**	VP	O/BY/CHA/2010	**RPSA**	Signal transmitting	Swine	Regulatory binding	Foot-and-Mouth Disease Virus Capsid Protein VP1 Interacts with Host Ribosomal Protein SA To Maintain Activation of the MAPK Signal Pathway and Promote Virus Replication	Zhu (2020) [85]
**VP1**	VP	N.D	**Sorcin**	Signal transmitting	Swine	N.D	Engagement of soluble resistance-related calcium binding protein (sorcin) with foot-and-mouth disease virus (FMDV) VP1 inhibits type I interferon response in cells	Li (2013) [86]
**VP1**	VP	O/BY/CHA/2010	**TPL2**	Signal transmitting	Swine	Polyubiquitination	Foot-and-Mouth Disease Virus Structural Protein VP1 Destroys the Stability of TPL2 Trimer by Degradation TPL2 to Evade Host Antiviral Immunity	Zhang (2020) [87]
**2C**	NS	O/BY/CHA/2010	**NOD2**	Viral sensing	Swine	Inhibition of protein expression	Foot-and-Mouth Disease Virus Antagonizes NOD2-Mediated Antiviral Effects by Inhibiting NOD2 Protein Expression	Liu (2019) [64]
**2C**	NS	O/BY/CHA/2010	**DDX21**	Viral sensing and Signal transmitting	Swine	Degradation via caspase pathway	DDX21, a Host Restriction Factor of FMDV IRES-Dependent Translation and Replication	Abdullah (2021) [65]
**2C**	NS	A/IND40/2000	**MARCHF7**	Signal transmitting	Swine	N.D	Identification of novel interactions between host and non-structural protein 2C of foot-and-mouth disease virus	Mahajan (2021) [88]
**2C**	NS	N.D	**Nmi**	Signal transmitting	Swine	Recruitment	A critical role of interferon-induced protein IFP35 in the type I interferon response in cells induced by foot-and-mouth disease virus (FMDV) protein 2C	Zeng (2014) [89]
**2C**	NS	O/BY/CHA/2010	**RIP2**	Signal transmitting	Swine	Inhibition of protein expression	Foot-and-Mouth Disease Virus Inhibits RIP2 Protein Expression to Promote Viral Replication	Liu (2021) [53]
**2C**	NS	N.D	**IFP35**	Signal transmitting and Transcription	Swine	Recruitment	A critical role of interferon-induced protein IFP35 in the type I interferon response in cells induced by foot-and-mouth disease virus (FMDV) protein 2C	Zeng (2014) [89]
**VP3**	VP	O/Taiwan/97	**TLR2**	Viral sensing	Human	Regulatory binding	Capsid proteins of foot-and-mouth disease virus interact with TLR2 and T CD14 to induce cytokine production	Lin (2020) [36]
**VP3**	VP	O/Taiwan/97	**CD14**	Signal transmitting	Human	Regulatory binding	Capsid proteins of foot-and-mouth disease virus interact with TLR2 and T CD14 to induce cytokine production	Lin (2020) [36]
**VP3**	VP	O/Tibet/CHA/2010	**MAVS**	Signal transmitting	Human	Inhibition of mRNA synthesis	The VP3 structural protein of foot-and-mouth disease virus inhibits the IFN-β signaling pathway	Li (2016) [90]
**VP3**	VP	O/Tibet/CHA/99	**JAK 1**	Response amplification	Human	Regulatory binding	Foot-and-mouth disease virus structural protein VP3 degrades Janus kinase 1 to inhibit IFN-γ signal transduction pathways	Li (2016) [91]
**VP3**	VP	O/Tibet/CHA/99	**JAK 2**	Response amplification	Human	Regulatory binding	Foot-and-mouth disease virus structural protein VP3 degrades Janus kinase 1 to inhibit IFN-γ signal transduction pathways	Li (2016) [91]
**VP0**	VP	O/Tibet/CHA/99	**PCBP2**	Signal transmitting	Swine	Regulatory binding	Poly (rC) binding protein 2 interacts with VP0 and increases the replication of the foot-and-mouth disease virus	Li (2019) [92]
**VP4**	VP	O/BY/CHA/2010	**NME1**	Signal transmitting	Swine	Degradation via macroautophagy pathway	Foot-and-mouth disease virus induces lysosomal degradation of NME1 to impair p53-regulated interferon-inducible antiviral genes expression	Feng (2018) [83]
**3B**	NS	O/BY/CHA/2010	**RIG-I**	Signal transmitting	Swine	Regulatory binding	Foot-and-Mouth Disease Virus 3B Protein Interacts with Pattern Recognition Receptor RIG-I to Block RIG-I-Mediated Immune Signaling and Inhibit Host Antiviral Response	Zhang (2020) [93]
**3D**	NS	A24/Cruzeiro/Brazil 1955	**Sam68**	Translation	Mouse	Regulatory binding	Analysis of the interaction between host factor Sam68 and viral elements during foot-and-mouth disease virus infections	Ray (2015) [94]
**3D**	NS	O/BY/CHA/2010	**DDX1**	N.D	Swine	Regulatory binding	The DEAD-Box RNA Helicase DDX1 Interacts with the Viral Protein 3D and Inhibits Foot-and-Mouth Disease Virus Replication	Xue (2019) [95]
